# MicroRNAs in cardiovascular disease: an introduction for clinicians

**DOI:** 10.1136/heartjnl-2013-305402

**Published:** 2015-03-26

**Authors:** Simon P R Romaine, Maciej Tomaszewski, Gianluigi Condorelli, Nilesh J Samani

**Affiliations:** 1 Department of Cardiovascular Sciences, University of Leicester, Leicester, UK; 2 NIHR Leicester Cardiovascular Biomedical Research Unit, Leicester, UK; 3 University of Milan, Milan, Italy

## Abstract

MicroRNAs (miRNAs) are small, non-coding, RNA molecules approximately 22 nucleotides in length which act as post-transcriptional regulators of gene expression. Individual miRNAs have been shown to regulate the expression of multiple genes. Conversely, the expression of individual genes can be regulated by multiple miRNAs. Consequently, since their discovery just over 20 years ago, miRNAs have been identified as key regulators of complex biological processes linked to multiple cardiovascular pathologies, including left ventricular hypertrophy, ischaemic heart disease, heart failure, hypertension and arrhythmias. Furthermore, since the finding that miRNAs are present in the circulation, they have been investigated as novel biomarkers, especially in the context of acute myocardial infarction (AMI) and heart failure. While there is little convincing evidence that miRNAs can outperform traditional biomarkers, such as cardiac troponins, in the diagnosis of AMI, there is potential for miRNAs to complement existing risk prediction models and act as valuable markers of post-AMI prognosis. Encouragingly, the concept of miRNA-based therapeutics is developing, with synthetic antagonists of miRNAs (antagomiRs) currently in phase II trials for the treatment of chronic hepatitis C virus infection. In the cardiovascular field, promising preclinical studies suggest that they could be useful in treating disorders ranging from heart failure to dyslipidaemia, although several challenges related to specificity and targeted delivery remain to be overcome. Through this review, we provide clinicians with a brief overview of the ever-expanding world of miRNAs.

## Introduction

Only 3% of the human genome codes for proteins. The remainder, previously thought to be ‘junk’, is now recognised to also be extensively transcribed.^w1^ While not converted into proteins, these non-coding RNAs which include short (approximately 22 nucleotides) microRNAs (miRNAs) and longer (>200 nucleotides) long non-coding RNAs (lncRNAs),^w2^ are now recognised to play important roles in gene regulation and function, thereby opening a new loop in the regulation of human biology.^1^ ^w3–w5^ Here we focus on miRNAs.

A strong body of evidence has been accumulated in recent years demonstrating a role for miRNAs in the pathogenesis of numerous cardiovascular diseases.^2^ Several excellent reviews provide an overview of miRNA biology,^1^ ^w4^ their involvement in cardiovascular biology and pathology^3^ ^4^ ^w3^ as well as their potential to become diagnostic and prognostic biomarkers of cardiovascular disease^2^ ^w6 w7^ and novel therapeutics agents.^5^ The purpose of this review, aimed primarily at clinicians, is to provide a brief overview of all of the above aspects of this fascinating topic.

## Biology of MicroRNAs

The existence of miRNAs is a relatively recent discovery. The first miRNA was discovered in 1993 and it was not until the turn of the century that they were identified in humans.^1^ ^w8–w10^ Since then, the field has exploded with current estimates suggesting that there are in excess of 2000 human miRNAs (catalogued in mirBase (http://www.mirbase.org)). During this time the nomenclature of miRNAs has evolved and can sometimes be confusing—we have explained the most common nomenclature in table 1. Similarly, explanations of key terms related to the miRNA world are given in box 1.

**Table 1:** Nomenclature of microRNAs (miRNAs)

Nomenclature format	Explanation
*mir* or miR	The genes that encode miRNAs, primary transcripts of miRNAs, and stem-loop precursor miRNAs are all named using the italicised prefix ‘*mir*’. Mature miRNAs are named using the non-italicised prefix ‘miR’.
*mir-X* or miR-X	With the exception of a few early miRNAs (such as the let family), miRNAs are sequentially assigned unique identifying numbers, depending on when they are first published, for example, *mir-31* or miR-31.
*mir-Xa*, *mir-Xb*, …	Similar miRNA sequences within a species are distinguished by a lettered suffix, for example, *mir-181a* and *mir-181b*. However, this does not imply shared targets or functions.
*mir-X-1*, *mir-X-2*, …	Identical miRNA sequences within a species are distinguished by a numerical suffix, for example, *mir-7-1* (located on chromosome 9), *mir-7-2* (located on chromosome 15) and *mir-7-3* (located on chromosome 19), can all produce identical mature miRNAs.
miR-X or miR-X*	Mature miRNAs can be formed from either arm of the stem–loop precursor miRNA (pre-miRNA; figure 1). In the majority of cases, one arm is much more commonly formed than the other. Previous convention was to name these strands according to their relative abundance, with the more common known as the ‘guide’ strand and taking the name miR-X (eg, miR-181a) and the less common form known as the ‘passenger’ strand and taking the name miR-X* (eg, miR-181a*).
miR-X-5p or miR-X-3p	The latest convention is to name mature miRNAs by the arm of the pre-miRNA from which they are derived, regardless of their abundance—those from the 5′ arm are named miR-X-5p and those from the 3′ arm as miR-X-3p. For example, miR-181a is now known as miR-181a-5p and miR-181a* is now known as miR-181a-3p. This avoids problems with the previous system if the abundance of each arm changes between tissues, developmental stages, or species.
hsa-miR-X, rno-miR-X, …	All of the above naming conventions can be preceded by a three-letter code which identifies the species the miRNA is from, for example, hsa=homo sapiens (human); rno=rattus norvegicus (rat). Therefore, miR-181a-5p found in humans, could be represented as hsa-miR-181a-5p. Identical miRNAs are given the same number, regardless of species, for example, hsa-miR-21 and rno-miR-21.

The nomenclature of miRNAs has changed over time and can often be confusing. This table explains some of the most common nomenclature formats, both previous and current. X, in the above formatting examples, is used as a generic term to represent the unique identifying number given to individual miRNAs.

Box 1 **Key terms**

*AntagomiRs or anti-miRs*: synthetic antagonists of microRNAs (miRNAs) that prevent endogenous miRNAs binding to their mRNA targets

**Figure 1:**
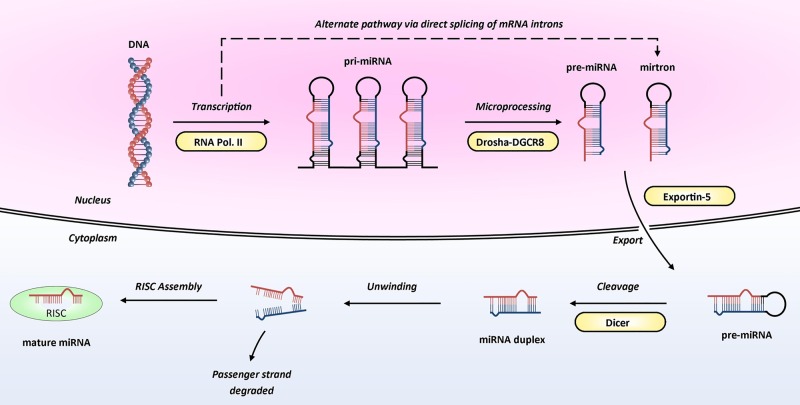
Biogenesis of microRNA. MicroRNA genes are transcribed by RNA polymerase II into molecules approximately 2 kb long called primary miRNAs (pri-miRNAs). Within the nucleus, these are cleaved into precursor miRNAs (pre-miRNAs) by Drosha, an RNase III enzyme, in association with DGCR8, an RNA-binding protein. Pre-miRNAs are approximately 60–100 nucleotides in length and have a hairpin structure. Interestingly, the presence of pre-miRNAs that are processed by direct splicing of introns (and thereby bypassing Drosha processing; dashed arrow) has also been reported; these are known as mirtrons.^w4^ Both pre-miRNAs and mirtrons are actively transported to the cytoplasm by the Ran-GTP dependent transporter, Exportin 5. Within the cytoplasm, pre-miRNAs are further cleaved by Dicer (another RNase III enzyme) generating unstable double-stranded miRNA duplexes—these duplexes are approximately 22 nucleotides in length and contain a functional miRNA ‘guide’ strand and a ‘passenger’ stand (previously termed miR-X*). Subsequently, the duplex is unwound and the passenger strand degraded, leaving the guide strand to enter the RNA-induced silencing complex (RISC) by associating with Argonaute proteins. Image adapted from Wienholds and Plasterk with permission from Elsevier.^30^

*Dicer*: an RNase enzyme with an essential role in miRNA processing, cleaving pre-miRNAs into miRNA duplexes (see figure 1)

*Drosha*: an RNase enzyme that, in combination with DGCR8, cleaves primary miRNAs into stem-loop precursor miRNAs (see figure 1)

*MicroRNAs*: small, non-coding, RNA molecules approximately 22 nucleotides in length which act as post-transcriptional regulators of gene expression

*MicroRNA mimics*: synthetic double-stranded RNAs that mimic endogenous miRNAs

*RISC (RNA-induced silencing complex)*: a multiprotein complex that uses the incorporated mature miRNA to guide it to target mRNA sites, where it is able to induce gene-silencing

**Figure 2:**
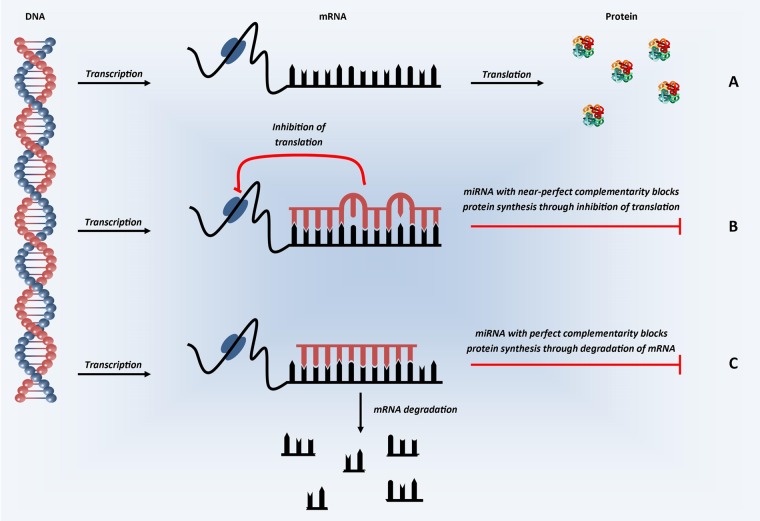
Schematic representation of microRNA mechanism of action. In the first step of protein synthesis, the DNA which codes for the protein of interest is converted into mRNA (transcription). (A) In the absence of miRNA, the mRNA transcripts are converted into protein (translation). (B) In the presence of miRNA with partial, near-perfect complementarity to the mRNA of interest, miRNA binds in the 3′ UTR and represses translation—inhibiting protein synthesis. (C) In the presence of miRNA with perfect complementarity, miRNA binding in the 3′ UTR is thought to inhibit protein synthesis through the induction of mRNA degradation. In humans, perfect complementarity is rare, with varying degrees of partial complementarity the predominant situation.

A basic schematic of how miRNAs are transcribed from the genome and processed into ≈22-mer mature (active) miRNAs is shown in figure 1. Mature miRNAs enter the RNA-induced silencing complex (RISC) by associating with Argonaute proteins. The targeting of the RISC to specific messenger RNA(s) is achieved by binding of the miRNA within the RISC to complementary sequences in the 3′ untranslated region (UTR) of target messenger RNA (mRNA). Once bound, the RISC is able to induce post-transcriptional gene silencing, through translational inhibition and/or mRNA degradation (figure 2), leading to the miRNA-mediated downregulation of the corresponding target protein.^1^ ^w3–w5^ The exact mechanisms by which targeting and silencing occur remain incompletely understood, although it is clear perfect complementarity across all 22 nucleotides is not required; indeed such a situation occurs rarely, if ever, in humans. It is also well recognised that pairing in the ‘seed’ region of the miRNA (nucleotides 2–7 or 8) appears most important for targeting mRNAs.^w11 w12^ Several software tools that predict which mRNAs may be targets for each miRNA sequence have been developed, including TargetScan (http://www.targetscan.org) and miRanda (http://www.microRNA.org). Experimentally validated targets have also been catalogued in TarBase (http://www.microrna.gr/tarbase).

As our knowledge of miRNA biogenesis has advanced, additional layers of complexity have been uncovered. For example, pathways that bypass Drosha (figure 1) or Dicer processing have been identified^6^ and a number of studies have shown a functional role for the less abundant (passenger) strands of some miRNA duplexes. These complexities of miRNA biogenesis are reviewed elsewhere.^6^ Furthermore, instances of RISC binding in the 5′ UTR of mRNA have been reported,^w13^ as have rare instances of miRNA mediated upregulation of protein synthesis.^w14^ Finally, the impact of single nucleotide polymorphisms (SNPs) in genes involved in miRNA biogenesis and processing, as well as SNPs in miRNA genes or their target sites, is beginning to be uncovered.^w15 w16^

Nevertheless, the key feature of the mechanism of action of miRNAs is that a single miRNA can regulate the expression of several genes, depending on the specificity of the target sequence; conversely, individual genes can be regulated by different miRNAs if they carry complementary sequences for more than one miRNA—these factors lead to a highly complex regulatory mechanism.^w3 w4^

## MicroRNAs in cardiovascular biology and disease

### MicroRNAs in the developing and healthy adult heart

miRNAs have been identified in cardiac tissue at all stages of development and those highly expressed in the foetal heart include miR-21, miR-29a, miR-129, miR-210, miR-211, miR-320, miR-423, and let-7c.^7^ The critical role of miRNAs during embryonic and postnatal cardiac development has been established by studies that have both globally^w17^ and cardioselectively^8^ depleted Dicer, an enzyme whose role is essential for miRNA processing (figure 1). This non-specific disruption of multiple miRNAs led to pericardial oedema, poorly developed ventricular myocardium and rapid lethality in mouse models.^8^ ^w17 w18^

In the healthy adult heart, data from a large sequencing project,^9^ along with other studies, has identified a number of miRNAs that are highly expressed in non-diseased cardiac tissue and thus likely to play a key role in both normal cardiac maintenance and disease.^1^ ^9^ ^10^ These include miR-1, miR-16, miR-27b, miR-30d, miR-126, miR-133, miR-143, miR-208 and the let-7 family.

### MicroRNAs, structural cardiac remodelling and heart failure

Cardiac remodelling allows the heart to adapt to external stressors. However, chronic activation of key remodelling processes, such as myocyte hypertrophy and fibrosis, becomes pathological and are a significant component of multiple cardiovascular diseases, including myocardial infarction (discussed in detail by Fiedler and Thum^w19^), cardiomyopathies and heart failure.^w20^ As with embryonic development, the overall role of miRNAs in the normal functioning of the adult heart has been demonstrated with *Dicer* deletion, which in adult mice led to biventricular enlargement and fibrosis, myocyte hypertrophy, induction of foetal transcriptome programme and sudden death.^8^ These findings implicate miRNAs as important regulators of cardiac remodelling.

Predictably, those miRNAs which are most abundant in cardiac tissue have been best linked to cardiac remodelling. These include miR-133 and miR-1, which are instrumental in skeletal myoblast proliferation and differentiation, respectively.^w21^ Indeed, Carè *et al*^11^ reported an inverse relationship between miR-133 and miR-1 expression and cardiac hypertrophy in both mouse and human tissues. This link was further supported using pharmacological techniques to modulate miRNA levels. AntagomiRs (also known as anti-miRs) are antisense oligonucleotides which sequester mature miRNA leading to functional inhibition.^w22^ Carè *et al*^11^ showed that a single infusion of a miR-133 antagomiR induced cardiac hypertrophy in mice.

More recently, therapeutic potential has been demonstrated through targeting miR-25 and its inhibition of the sarcoplasmic reticulum calcium uptake pump, SERCA2a.^12^ Wahlquist *et al*^12^ demonstrated that increased miR-25 levels can depress cardiac function and more importantly, inhibition of miR-25 using antagomiR technology effectively restored cardiac function and significantly improved survival (compared with control antagomiR treated mice) in a mouse model of heart failure. Additionally, the authors demonstrated an anti-miR-25 mediated reduction in fibrosis and normalisation of cardiomyocyte size. Interestingly, recent work by Bang *et al*^13^ has demonstrated that miRNAs secreted by cardiac fibroblasts may also act as paracrine mediators of cardiomyocyte hypertrophy.

### MicroRNAs and atherosclerosis

The initiation and progression of atherosclerosis are complicated, multifaceted pathologies which remain incompletely understood. Key processes include endothelial cell (EC) dysfunction, infiltration of inflammatory cells, lipid dysregulation and vascular smooth muscle cell (VSMC) differentiation. Specific miRNAs have been shown to influence each of these processes (see table 2 and online supplementary table S1).

**Table 2:** Summary of microRNAs (miRNAs) and their targets that have been associated with processes fundamental to the initiation and progression of atherosclerosis*

miR	Target(s)	miR	Target(s)
Cholesterol metabolism and homeostasis			
miR-10b	ABCA1, ABCG1	miR-185	SREBP-2
miR-26	ABCA1, ARL7	miR-206	LXRα
miR-27a/b	ABCA1, ACAT1, ANGPTL3, CD36, GPAM, LPL	miR-378	ABCG1
miR-30c	LPGAT1, MTP	miR-467b	LPL
miR-33a/b	ABCA1, ABCG1, AMPKα, CPT1A, CROT, HADHB, IRS2, NPC1, PRKAA1, SREBP-1	miR-613	ABCA1, LXRα
miR-106b	ABCA1	miR-758	ABCA1
miR-144	ABCA1		
Endothelial cell dysfunction			
miR-1	MLCK	miR-221/222	c-Kit, eNOS, ETS-1, PAK1, p27, p57, STAT5A
miR-27a/b	SEMA6A	miR-223	IGF-1R
miR-34a	SIRT1	miR-365	BCL-2
miR-92a	KLF2, KLF4, PTEN, SOCS5	miR-492	Resistin
miR-126-5p	DLK1	miR-513a-5p	XIAP
miR-144	IDH2	miR-663	-
miR-146a	NOX4	miR-712	TIMP3
miR-155	AT1R, ETS-1, MLCK	let-7c	BCL-XL
miR-216a	BECN1	let-7g	CASP3, SMAD2, TGFBR1, THBS1
miR-217	SirT1		
Inflammation			
miR-9	ACAT1, PPARδ	miR-126-3p	VCAM-1
miR-10a	MAP3K7, βTRC	miR-145	JAM-A
miR-15a	CARM1	miR-146a/b	CD40L, IRAK1, IRAK2, TLR4, TRAF6
miR-17-3p	ICAM-1	miR-147	–
miR-21	PPARα, TLR4	miR-155	BCL-2, ETS-1, FADD, HBP1, MAP3K10
miR-29a	LPL	miR-181a	c-Fos
miR-31	E-selectin	miR-181b	IPOA3
miR-125a-5p	ORP9	miR-342-5p	AKT1
Vascular smooth muscle cell differentiation and proliferation			
miR-1	KLF4, MRTF-A, PIM-1	miR-181a	OPN
miR-21	BCL-2, PDCD4, PPARα, PTEN, TPM1	miR-195	CDC42
miR-26a	SMAD1, SMAD4	miR-208	p21
miR-29b	DNMT3b	miR-221/222	c-Kit, p27, p57
miR-125b	SP7	miR-490-3p	PAPP-A
miR-126	BCL-2, FOXO3, IRS1	miR-638	NOR1
miR-132	LRRFIP1	miR-663	JUNB, MYL9
miR-133	SP1	let-7d	KRAS
miR-133a	IGF-1R, RUNX2	let-7g	LOX-1
miR-143/145	ELK1, fascin, KLF4, KLF5, PDGF-Rα, PKC-ε		

* A fully referenced version of this table can be found as online supplementary table S1.

ABCA1, ATP binding cassette transporter A1; ABCG1, ATP binding cassette transporter G1; ACAT1, acyl-CoA:cholesterol acyltransferase 1; AKT1, v-akt murine thymoma viral oncogene homologue 1; AMPKα, AMP kinase subunit-α; ANGPTL3, angiopoietin-like 3; ARL7=ADP-ribosylation factor-like 7; AT1R, angiotensin II type 1 receptor; BCL-XL, B-cell lymphoma-extra large; BCL-2=B-cell lymphoma 2; BCL-6, B-cell lymphoma 6; BECN1, Beclin1; CARM1, coactivator-associated arginine methyltransferase 1; CASP3, caspase-3; CDC42, cell division control protein 42; CD36, scavenger receptor CD36; CPT1A, carnitine palmitoyltransferase 1A; CROT, carnitine O-octaniltransferase; DLK1, delta-like 1 homologue; DNMT3b, DNA methyltransferase 3b; ELK1=ELK1, member of ETS oncogene family; eNOS, endothelial nitric oxide synthase; ETS-1, E26 transformation-specific sequence 1; FADD, Fas-associated death domain-containing protein; FOXO3, forkhead box O3; GPAM, glycerol-3-phosphate acyltransferase 1; HADHB, hydroxyacyl-CoA-dehydrogenase; HBP1, HMG box-transcription protein 1; ICAM-1, intercellular adhesion molecule 1; IDH2, isocitrate dehydrogenase 2; IGF-1R, insulin like growth factor 1 receptor; IPOA3, importin-α3; IRAK1, interleukin-1 receptor-associated kinase 1; IRAK2, interleukin-1 receptor-associated kinase 2; IRS1, insulin receptor substrate 1; IRS2, insulin receptor substrate 2; JAM-A, junctional adhesion molecule-A; JUNB, transcription factor Jun-B; KLF2, Krüppel-like factor 2; KLF4, Krüppel-like factor 4; KRAS, Kirsten rat sarcoma viral oncogene homologue; LOX-1, lectin-like oxidised LDL receptor-1; LPGAT1, lysophosphatidylglycerol acyltransferase 1; LPL, lipoprotein lipase; LRRFIP1, leucine-rich repeat (in Flightless 1) interacting protein-1; LXRα, liver X receptor α; MAP3K7, mitogen-activated kinase kinase kinase 7; MAP3K10, mitogen-activated kinase kinase kinase 10; MLCK, myosin light chain kinase; MYL9, myosin light chain 9; MRTF-A, myocardin-related transcription factor A; MTP, microsomal triglyceride transfer protein; NOR1, neuron-derived orphan receptor 1; NOX4, NADPH oxidase 4; NPC1, Niemann-Pick C1; OPN, osteopontin; ORP9, oxysterol binding protein-like 9; PAK1, p21/Cdc42/Rac1-activated kinase 1; PAPP-A, pregnancy-associated plasma protein A; PDCD4=programmed cell death 4; PDGF-Rα, platelet-derived growth factor receptor α; PKC-ε, protein kinase C- ε; PIM-1, serine/threonine-protein kinase PIM-1; PPARα, peroxisome proliferators-activated receptor-α; PPARδ, peroxisome proliferators-activated receptor-δ; PRKAA1, protein kinase, AMP-activated, α 1 catalytic subunit; PTEN, phosphatase and tensin homologue; RUNX2, Runt-related transcription factor 2; SEMA6A, semaphorin 6A; SIRT1, sirtuin 1; SirT1, silent information regulator 1; SMAD1, SMAD family member 1; SMAD2, SMAD family member 2; SMAD4, SMAD family member 4; SOCS5, suppressor of cytokine signalling 5; SP1, SP1 transcription factor; SP7, SP7 transcription factor; SREBP-1, sterol regulatory element-binding protein 1; SREBP-2, sterol regulatory element-binding protein 2; STAT5A, signal transducer and activator of transcription 5A; TGFBR1, transforming growth factor beta receptor 1; THBS1, thrombospondin 1; TIMP3, tissue inhibitor of metalloproteinase 3; TLR4, toll-like receptor 4; TPM1, tropomyosin 1; TRAF6, TNF receptor associated factor 6; VCAM-1, vascular cell adhesion molecule 1; XIAP, X-linked inhibitor of apoptotic protein; βTRC, β-transducin repeat-containing.

Endothelial cell dysfunction is a key step in the initiation of atherosclerosis. The predilection for plaque formation at sites of arterial branching or bifurcation has identified disturbed laminar flow as a predisposing factor to EC dysfunction. Schober *et al*^14^ demonstrated that miR-126-5p is critical in maintaining a proliferative reserve of ECs in response to shear stress through the suppression of delta-like homologue 1 (Dlk1) and that diminished levels of miR-126-5p reduced the proliferative reserve of ECs, promoting plaque formation. Additionally, increased permeability of ECs leads to the infiltration of inflammatory cells and miRNA-155 has been shown to be play a key role in this process.^w23 w24^

In response to inflammatory cell infiltration, VSMC migration from the media to the intima is another key process in atherosclerosis. A number of miRNAs have been linked to this phenotype. For example, the miR-143/145 complex has been shown as a critical regulator of VSMC differentiation.^w25^ Interestingly, Lovren *et al*^15^ were able to demonstrate reduced atherosclerotic plaque formation in ApoE knockout mice treated with VSMC-targeted miR-145 therapy, and observed features of increased plaque stability.

### MicroRNAs and arrhythmias

Atrial fibrillation (AF) is the most common arrhythmia, especially among elderly populations, and is the end-stage manifestation of multiple pathological changes, including both structural and electrical remodelling.^16^ Early work demonstrated that miR-1 levels were markedly reduced (≈86%) in atrial tissue from patients with AF compared with those without; a possible effect of miR-1 on inward-rectifier K^+^ currents (I_K1_) was postulated.^w26^ Lu *et al*^17^ demonstrated that the overexpression of miR-328 enhanced AF vulnerability and knockdown of miR-328 reduced AF vulnerability in mouse models, and reported 3.5-fold elevation of miR-328 levels in atrial samples from patients with AF versus those without; miR-223 and miR-664 were also elevated but interestingly, miR-1 was unaltered. Despite this, miR-1 has also been implicated in the modulation of a wide variety of Ca^2+^ handling proteins^16^ and has been linked to ventricular arrhythmias.^w27 w28^

### MicroRNAs and hypertension

Several studies have highlighted the involvement of miRNAs in blood pressure regulation, particularly by affecting the renin-angiotensin-aldosterone system. Marques *et al*^18^ compared both miRNA and mRNA expression in renal tissue from untreated hypertensive and normotensive individuals. Using microarray technology, they screened 850 miRNAs and identified nine which exhibited expression differences, with two of the miRNAs (miR-181a and miR-663) linked to repression of renin expression in the human kidney. Eskildsen *et al* reported that miR-132 and miR-212 were upregulated in the heart, aorta and kidneys of rats who received a 10-day infusion of angiotensin-II, and downregulated in the internal mammary artery of patients treated with angiotensin-II receptor type 1 blockers, suggesting a role for miR-132 and miR-212 in angiotensin-II mediated hypertension.^w29^

Boettger *et al*^19^ demonstrated that miR-143/145 knockout mice exhibited significantly reduced blood pressure compared with wild-type littermates and identified angiotensin converting enzyme (ACE) mRNA as a target for miR-145. Furthermore, Santovito *et al*^w30^ found that miR-145 was overexpressed in atherosclerotic plaques of hypertensive versus normotensive patients undergoing carotid endarterectomy, implicating miR-145 as a potentially key regulator of blood pressure mediated vascular damage. A more extensive review of miRNAs and hypertension has recently been published.^20^

## Circulating MicroRNAs in cardiovascular disease

In 2008, the presence of miRNA was detected in human plasma and serum.^21^ ^w31^ In their landmark paper, Mitchell *et al*^21^ demonstrated robust stability of such circulating miRNAs to storage at room temperature, repeated freeze–thaw cycles and degradation by endogenous RNase activity. Critically, when synthetic miRNAs were added to plasma prior to an RNase denaturing solution, they were rapidly degraded—suggesting that circulating miRNAs are somehow protected from endogenous RNase activity. Explanations for this include the localisation of miRNAs to microvesicles and the presence of miRNA-protein/lipoprotein complexes in extracellular fluids.^w7^ Subsequently, miRNAs have been detected in a wide range of bodily fluids, including urine, saliva, cerebrospinal fluid and breast milk.^w32^ This has allowed researchers to compare circulating miRNA levels between patients and healthy controls and assess their utility as diagnostic or prognostic biomarkers.

### Acute coronary syndromes

**Figure 3:**
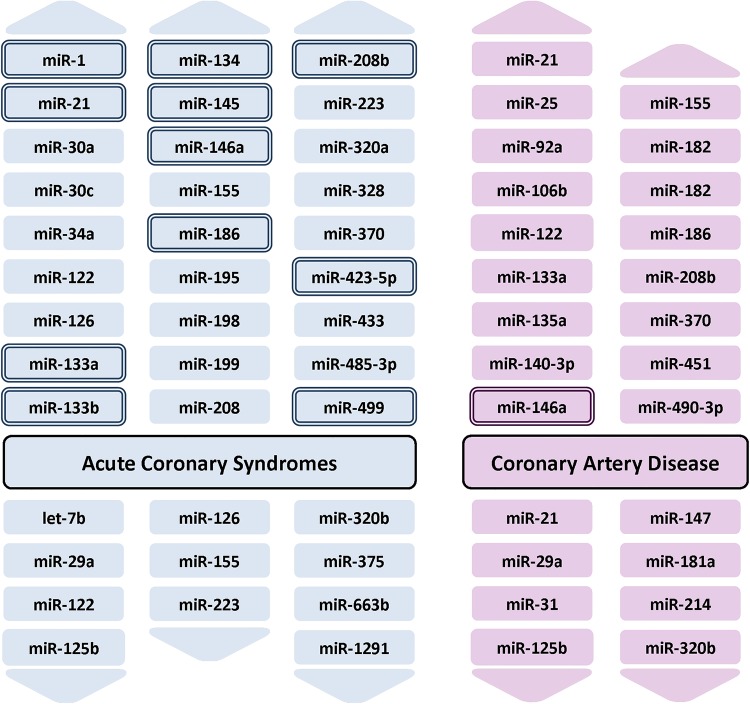
Circulating microRNAs associated with acute coronary syndromes and coronary artery disease. MicroRNAs with a double border have been linked to the associated trait by more than one study—for details online supplementary tables S2 and S4.

Several studies have investigated the role of miRNAs in identifying patients with acute myocardial infarction (AMI; see online supplementary table S2). Although a number of promising candidates have emerged including miR-1, miR-133a, miR-133b, miR-208a, miR-499, miR-499-5p (figure 3), further validation is required for most of them.

In the largest study conducted to date, Devaux *et al*^22^ measured the levels of six miRNAs in 1155 patients attending the emergency department with acute chest pain, of which 224 were diagnosed with AMI. While the levels of miR-208b, miR-499 and miR-320a were significantly higher in patients with AMI compared with other diagnoses, none were able to out-perform cardiac Troponin T (cTnT) or high-sensitivity cTnT (hsTnT), or add to their diagnostic ability.

This highlights a major obstacle in the identification of miRNAs as diagnostic biomarkers of AMI. As the diagnostic criteria for AMI place significant emphasis on cardiac troponins, it becomes extremely difficult, if not impossible, for any new biomarkers to outperform them. While some have proposed that miRNAs may be able to act as biomarkers in the *earlier* diagnosis of AMI, prior to the detection of troponin,^w33^ this remains to be established. Furthermore, such use of miRNAs as biomarkers in acute coronary syndromes is currently also limited by the time taken to investigate the level of candidate miRNAs in a single sample. At present, this cannot be achieved within a timeframe of clinical utility. However, the role of miRNAs as diagnostic biomarkers in less time-sensitive diagnoses and also their role as *prognostic* biomarkers may herald more potential (see online supplementary table S3).

Unfortunately, Devaux *et al*^22^ failed to find support for this suggestion in the context in AMI. They reported that while miR-208b moderately predicted survival at 30 days, none of the miRNAs were able to predict long-term mortality (two years).

### Coronary artery disease

More encouraging results have been reported away from the diagnosis of AMI. A number of miRNAs have been linked to a diagnosis of coronary artery disease (see figure 3 and online supplementary table S4). More interestingly, Zampetaki *et al*^23^ identified three miRNAs (miR-126, miR-197, and miR-223) that were predictive of incident AMI in a prospective study of cardiovascular disease (with 10-year follow-up). Furthermore, the addition of these three miRNAs to the Framingham Risk Score improved classification to a greater degree than hsTnT. While further validation is required, these results suggest a potential role for miRNAs in predicting risk of future AMI.

### Heart failure

**Figure 4:**
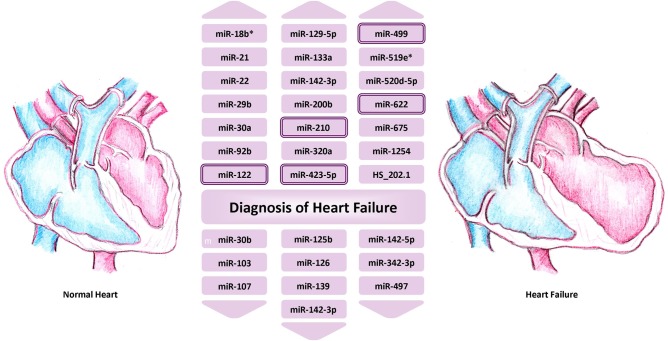
Circulating microRNAs associated with a diagnosis of heart failure. MicroRNAs with a double border have been linked to heart failure by more than one study—for details online supplementary table S5.

As with acute coronary syndromes and coronary artery disease, early studies investigating the role of circulating miRNAs in heart failure used small sample numbers and generated a list of potential miRNAs with altered circulating levels in patients with heart failure—these include miR-122, miR-210, miR-423-5p, miR-499 and miR-622 (see figure 4 and online supplementary table S5).

Additional work has shown potential for using miRNAs as prognostic indicators in the development of heart failure. Squire and colleagues reported that a panel of four miRNAs (miR-16, miR-27a, miR-101, miR-150) improved the prediction of left ventricle (LV) contractility six months after AMI, in a multivariable model that included Nt-proBNP, an established biomarker of heart failure.^24^ These findings built upon their previous demonstration that reduced levels of miR-150 levels were associated with increased LV remodelling after ST segment elevation myocardial infarction (STEMI),^w34^ and herald promise in the use of miRNAs, or panels of miRNAs, as diagnostic and prognostic predictors of heart failure.

Nevertheless, despite promising results, it should be stressed that the appropriate detection and quantification of circulating miRNAs is not without its problems. These have been nicely summarised by Zampetaki and Mayr^25^ and include difficulties in measuring absolute levels of miRNAs (as opposed to relative expression vs controls), the lack of standardised protocols (including endogenous ‘normalisation’ controls) and intra- and inter-laboratory variation. Finally, a problem of major relevance to samples taken during AMI or in other hospitalised patients is that heparin has been shown to be a significant inhibitor of PCR-based reactions (a crucial component of miRNA analysis).^w35^ Consequently, while research continues to identify potential miRNA biomarkers, it is essential that robust protocols and technical advances are made before they can be realistically introduced to the clinical environment.

## MicroRNA-based therapeutics

Given their key function in gene regulation, miRNAs provide promising therapeutic targets. This potential has been demonstrated in animal models,^12^ ^26^ ^27^ with the greatest progress (in cardiovascular disease) being made in treating hypercholesterolaemia. For example, miR-33a and miR-33b have been shown to regulate the sterol regulatory element-binding proteins, along with ATP-binding cassette (ABC) transporters ABCA1 and ABCG1 to control cholesterol transport.^27^ ^w36^ Encouragingly, this link has not only been shown in vitro, but also in mice models.^27^ Furthermore, Rayner *et al*^28^ showed that the inhibition of miR-33a and miR-33b in non-human primates led to sustained elevation of plasma high-density lipoprotein cholesterol (up to 50%) and concomitant reductions of very-low-density lipoprotein cholesterol with no apparent adverse effects. Although no significant changes in total or low-density lipoprotein cholesterol were observed, the potential for such therapy in the treatment of dyslipidaemia and atherosclerosis heralds considerable potential.

However, these have yet to progress to human clinical trials. Encouragingly, this is beginning to happen in other fields—the first candidate to enter clinical trials is miravirsen (SPC3649) in the treatment of chronic hepatitis C virus (HCV) infection. miR-122 is essential to the stability and propagation of HCV RNA and miravirsen, an antagomiR of miR-122, can sequester and therefore inhibit miR-122.^29^ The results of the first phase II trial of miravirsen were published in 2013, reporting that five weekly injections resulted in a dose-dependent reduction in HCV RNA levels that lasted up to 10 weeks beyond the active treatment phase.^29^ Importantly, the initial safety and side effect profile was excellent.

Despite these promising results, the specialised chemistries required to implement miRNA therapeutics, such as 2-O-methyl-group-modified oligonucleotides and locked nucleic acid-modified oligonucleotides, remain complex. These chemistries and the challenging issues surrounding specificity, efficacy and safety of delivery mechanisms are nicely summarised by van Rooij *et al*.^5^ ^w37 w38^

## Future directions and concluding remarks

The exciting world of miRNA-based research and potential clinical application is developing rapidly. In just over a decade since their first discovery in humans, miRNAs have already provided important novel insights into the biology of several cardiovascular diseases. However, there remains much to learn. From a research perspective, further studies are required to elucidate the exact methods by which miRNAs are able to repress translation and initiate mRNA degradation. Similarly, the exact sources, location and role (eg, in cell to cell communication) of circulating miRNAs need to be better defined.^w6^

From a clinical perspective, miRNAs may provide valuable diagnostic and prognostic biomarkers. However, larger trials are required to establish whether current candidates offer additional benefit, over and above those of existing biomarkers of cardiovascular disease. Additionally, technological advances are required to enable rapid, reliable and reproducible results for the absolute quantification of circulating miRNAs to facilitate transition into clinical practice. Finally, while there remain a number of challenges to overcome, clinicians should be aware of the potential arrival of miRNA-based therapeutics into the domain of clinical medicine.

## Supplementary Material

Web supplement
