# Supplementary material for: MicroRNAs in cardiovascular disease: an introduction for clinicians
Source: Heart. 2015 Mar 26;101(12):921–8. doi: 10.1136/heartjnl-2013-305402 (PMC4484262; doi:10.1136/heartjnl-2013-305402)
Supplement: Web supplement [file heartjnl-2013-305402-s1.pdf]

SUPPLEMENTARY REFERENCES

ONLINE ONLY REFERENCES FOR MAIN TEXT

w1. Djebali S, Davis CA, Merkel A, et al. Landscape of transcription in human cells. *Nature* 2012;489:101-8.

w2. Thum T. Noncoding RNAs and myocardial fibrosis. *Nat Rev Cardiol* 2014;11:655-63.

w3. Batkai S, Thum T. MicroRNAs in hypertension: mechanisms and therapeutic targets. *Curr Hypertens Rep* 2012;14:79-87.

w4. Bauersachs J, Thum T. Biogenesis and regulation of cardiovascular microRNAs. *Circ Res* 2011;109:334-47.

w5. Ruby JG, Jan CH, Bartel DP. Intronic microRNA precursors that bypass Drosha processing. *Nature* 2007;448:83-6.

w6. van Empel VP, De Windt LJ, da Costa Martins PA. Circulating miRNAs: reflecting or affecting cardiovascular disease? *Curr Hypertens Rep* 2012;14:498-509.

w7. Zampetaki A, Willeit P, Drozdov I, et al. Profiling of circulating microRNAs: from single biomarkers to re-wired networks. *Cardiovasc Res* 2012;93:555-62.

w8. Lee RC, Feinbaum RL, Ambros V. The C. elegans heterochronic gene lin-4 encodes small RNAs with antisense complementarity to lin-14. *Cell* 1993;75:843-54.

w9. Pasquinelli AE, Reinhart BJ, Slack F, et al. Conservation of the sequence and temporal expression of let-7 heterochronic regulatory RNA. *Nature* 2000;408:86-9.

w10. Wightman B, Ha I, Ruvkun G. Posttranscriptional regulation of the heterochronic gene lin-14 by lin-4 mediates temporal pattern formation in C. elegans. *Cell* 1993;75:855-62.

w11. Brennecke J, Stark A, Russell RB, et al. Principles of microRNA-target recognition. *PLoS Biol* 2005;3:e85.

w12. Schirle NT, Sheu-Gruttadauria J, MacRae IJ. Gene regulation. Structural basis for microRNA targeting. *Science* 2014;346:608-13.

w13. Da Sacco L, Masotti A. Recent Insights and Novel Bioinformatics Tools to Understand the Role of MicroRNAs Binding to 5' Untranslated Region. *Int J Mol Sci* 2012;14:480-95.

w14. Vasudevan S, Tong Y, Steitz JA. Switching from repression to activation: microRNAs can up-regulate translation. *Science* 2007;318:1931-4.

w15. Gong J, Tong Y, Zhang HM, et al. Genome-wide identification of SNPs in microRNA genes and the SNP effects on microRNA target binding and biogenesis. *Hum Mutat* 2012;33:254-63.

w16. Zhang K, Deacon DC, Rao F, et al. Human heart rate: heritability of resting and stress values in twin pairs, and influence of genetic variation in the adrenergic pathway at a microribonucleic acid (microrna) motif in the 3'-UTR of cytochrome b561 [corrected]. *J Am Coll Cardiol* 2014;63:358-68.

w17. Bernstein E, Kim SY, Carmell MA, et al. Dicer is essential for mouse development. *Nat Genet* 2003;35:215-7.

w18. Zhao Y, Ransom JF, Li A, et al. Dysregulation of cardiogenesis, cardiac conduction, and cell cycle in mice lacking miRNA-1-2. *Cell* 2007;129:303-17.

w19. Fiedler J, Thum T. MicroRNAs in myocardial infarction. *Arterioscler Thromb Vasc Biol* 2013;33:201-5.

w20. Orenes-Piñero E, Montoro-García S, Patel JV, et al. Role of microRNAs in cardiac remodelling: New insights and future perspectives. *Int J Cardiol* 2013;167:1651-59.

w21. Chen JF, Mandel EM, Thomson JM, et al. The role of microRNA-1 and microRNA-133 in skeletal muscle proliferation and differentiation. *Nat Genet* 2006;38:228-33.

w22. Stenvang J, Petri A, Lindow M, et al. Inhibition of microRNA function by antimiR oligonucleotides. *Silence* 2012;3:1.

w23. O'Connell RM, Taganov KD, Boldin MP, et al. MicroRNA-155 is induced during the macrophage inflammatory response. *Proc Natl Acad Sci U S A* 2007;104:1604-9.

w24. Wei Y, Nazari-Jahantigh M, Chan L, et al. The microRNA-342-5p fosters inflammatory macrophage activation through an Akt1- and microRNA-155-dependent pathway during atherosclerosis. *Circulation* 2013;127:1609-19.

w25. Cordes KR, Sheehy NT, White MP, et al. miR-145 and miR-143 regulate smooth muscle cell fate and plasticity. *Nature* 2009;460:705-10.

w26. Girmatsion Z, Biliczki P, Bonauer A, et al. Changes in microRNA-1 expression and IK1 up-regulation in human atrial fibrillation. *Heart Rhythm* 2009;6:1802-9.

w27. Curcio A, Torella D, Iaconetti C, et al. MicroRNA-1 downregulation increases connexin 43 displacement and induces ventricular tachyarrhythmias in rodent hypertrophic hearts. *PLoS One* 2013;8:e70158.

w28. Yang B, Lin H, Xiao J, et al. The muscle-specific microRNA miR-1 regulates cardiac arrhythmogenic potential by targeting GJA1 and KCNJ2. *Nat Med* 2007;13:486-91.

w29. Eskildsen TV, Jeppesen PL, Schneider M, et al. Angiotensin II Regulates microRNA-132/-212 in Hypertensive Rats and Humans. *Int J Mol Sci* 2013;14:11190-207.

w30. Santovito D, Mandolini C, Marcantonio P, et al. Overexpression of microRNA-145 in atherosclerotic plaques from hypertensive patients. *Expert Opin Ther Targets* 2013;17:217-23.

w31. Lawrie CH, Gal S, Dunlop HM, et al. Detection of elevated levels of tumour-associated microRNAs in serum of patients with diffuse large B-cell lymphoma. *Br J Haematol* 2008;141:672-5.

w32. Weber JA, Baxter DH, Zhang S, et al. The microRNA spectrum in 12 body fluids. *Clin Chem* 2010;56:1733-41.

w33. Wang GK, Zhu JQ, Zhang JT, et al. Circulating microRNA: a novel potential biomarker for early diagnosis of acute myocardial infarction in humans. *Eur Heart J* 2010;31:659-66.

w34. Devaux Y, Vausort M, McCann GP, et al. MicroRNA-150: a novel marker of left ventricular remodeling after acute myocardial infarction. *Circ Cardiovasc Genet* 2013;6:290-8.

w35. Kaudewitz D, Lee R, Willeit P, et al. Impact of intravenous heparin on quantification of circulating microRNAs in patients with coronary artery disease. *Thromb Haemost* 2013;110:609-15.

w36. Marquart TJ, Allen RM, Ory DS, et al. miR-33 links SREBP-2 induction to repression of sterol transporters. *Proc Natl Acad Sci U S A* 2010;107:12228-32.

w37. van Rooij E, Olson EN. MicroRNA therapeutics for cardiovascular disease: opportunities and obstacles. *Nature Reviews: Drug Discovery* 2012;11:860-72.

w38. van Rooij E, Kauppinen S. Development of microRNA therapeutics is coming of age. *EMBO Mol Med* 2014;6:851-64.

SUPPLEMENTARY TABLES

Table S1: Summary of microRNAs and their targets that have been associated with processes fundamental to the initiation and progression of atherosclerosis

miR	Target(s)	Ref(s)
Cholesterol Metabolism & Homeostasis		
miR-10b	ABCA1, ABCG1	[1]
miR-26	ABCA1, ARL7	[2]
miR-27a/b	ABCA1, ACAT1, ANGPTL3, CD36, GPAM, LPL	[3,4]
miR-30c	LPGAT1, MTP	[5]
miR-33a/b	ABCA1, ABCG1, AMPKα, CPT1A, CROT, HADHB, IRS2, NPC1, PRKAA1, SREBP-1	[6-14]
miR-106b	ABCA1	[15]
miR-144	ABCA1	[16,17]
miR-185	SREBP-2	[18]
miR-206	LXRα	[19]
miR-378	ABCG1	[20]
miR-467b	LPL	[21,22]
miR-613	ABCA1, LXRα	[23]
miR-758	ABCA1	[24]
Endothelial Cell Dysfunction		
miR-1	MLCK	[25]
miR-27a/b	SEMA6A	[26]
miR-34a	SIRT1	[27]
miR-92a	KLF2, KLF4, PTEN, SOCS5	[28-31]
miR-126-5p	DLK1	[32]
miR-144	IDH2	[33]
miR-146a	NOX4	[34]
miR-155	AT1R, ETS-1, MLCK	[35,36]
miR-216a	BECN1	[37]
miR-217	SirT1	[38]
miR-221/222	c-Kit, eNOS, ETS-1, PAK1, p27, p57, STAT5A	[36,39-43]
miR-223	IGF-1R	[44]
miR-365	BCL-2	[45]
miR-492	Resistin	[46]
miR-513a-5p	XIAP	[47]
miR-663	-	[48]
miR-712	TIMP3	[49]
let-7c	BCL-XL	[50]
let-7g	CASP3, SMAD2, TGFBR1, THBS1	[51,52]
Inflammation		
miR-9	ACAT1, PPARδ	[53,54]
miR-10a	MAP3K7, βTRC	[55]
miR-15a	CARM1	[56]
miR-17-3p	ICAM-1	[57]
miR-21	PPARα, TLR4	[58,59]
miR-29a	LPL	[60]

miR	Target(s)	Ref(s)
Inflammation (continued)		
miR-31	E-selectin	[57]
miR-125a-5p	ORP9	[61]
miR-126-3p	VCAM-1	[62,63]
miR-145	JAM-A	[64]
miR-146a/b	CD40L, IRAK1, IRAK2, TLR4, TRAF6	[65-67]
miR-147	-	[68]
miR-155	BCL-2, ETS-1, FADD, HBP1, MAP3K10	[36,69-74]
miR-181a	c-Fos	[75]
miR-181b	IPOA3	[76]
miR-342-5p	AKT1	[77]
VSMC Differentiation & Proliferation		
miR-1	KLF4, MRTF-A, PIM-1	[78-80]
miR-21	BCL-2, PDCD4, PPARα, PTEN, TPM1	[81-84]
miR-26a	SMAD1, SMAD4	[85]
miR-29b	DNMT3b	[86]
miR-125b	SP7	[87]
miR-126	BCL-2, FOXO3, IRS1	[88]
miR-132	LRRFIP1	[89]
miR-133	SP1	[90]
miR-133a	IGF-1R, RUNX2	[91,92]
miR-143/145	ELK1, fascin, KLF4, KLF5, PDGF-Rα, PKC-ε	[93-99]
miR-181a	OPN	[100]
miR-195	CDC42	[101]
miR-208	p21	[102]
miR-221/222	c-Kit, p27, p57	[43,103-105]
miR-490-3p	PAPP-A	[106]
miR-638	NOR1	[107]
miR-663	JUNB, MYL9	[108]
let-7d	KRAS	[109]
let-7g	LOX-1	[110]

ABCA1 = ATP binding cassette transporter A1; ABCG1 = ATP binding cassette transporter G1; ACAT1 = acyl-CoA:cholesterol acyltransferase 1; AKT1 = v-akt murine thymoma viral oncogene homolog 1; AMPKα = AMP kinase subunit-α; ANGPTL3 = angiopoietin-like 3; ARL7 = ADP-ribosylation factor-like 7; AT1R = angiotensin II type 1 receptor; BCL-XL = B-cell lymphoma-extra large; BCL-2 = B-cell lymphoma 2; BCL-6 = B-cell lymphoma 6; BECN1 = Beclin1; CARM1 = coactivator-associated arginine methyltransferase 1; CASP3 = caspase-3; CDC42 = cell division control protein 42; CD36 = scavenger receptor CD36; CPT1A = carnitine palmitoyltransferase 1A; CROT = carnitine O-octaniltransferase; DLK1 = delta-like 1 homolog; DNMT3b = DNA methyltransferase 3b; ELK1 = ELK1, member of ETS oncogene family; eNOS = endothelial nitric oxide synthase; ETS-1 = E26 transformation-specific sequence 1; FADD = Fas-associated death domain-containing protein; FOXO3 = forkhead box O3; GPAM = glycerol-3-phosphate acyltransferase 1; HADHB = hydroxyacyl-CoA-dehydrogenase; HBP1 = HMG box-transcription protein 1; ICAM-1 = intercellular adhesion molecule 1; IDH2 = isocitrate dehydrogenase 2; IGF-1R = insulin like growth factor 1 receptor; IPOA3 = importin-α3; IRAK1 = interleukin-1 receptor-associated kinase 1; IRAK2 = interleukin-1 receptor-associated kinase 2;

IRS1 = insulin receptor substrate 1; IRS2 = insulin receptor substrate 2; JAM-A = junctional adhesion molecule-A; JUNB = transcription factor Jun-B; KLF2 = Krüppel-like factor 2; KLF4 = Krüppel-like factor 4; KRAS = Kirsten rat sarcoma viral oncogene homolog; LOX-1 = lectin-like oxidized LDL receptor-1; LPGAT1 = lysophosphatidylglycerol acyltransferase 1; LPL = lipoprotein lipase; LRRFIP1 = leucine-rich repeat (in Flightless 1) interacting protein-1; LXRα = liver X receptor α; MAP3K7 = mitogen-activated kinase kinase kinase 7; MAP3K10 = mitogen-activated kinase kinase kinase 10; MLCK = myosin light chain kinase; MYL9 = myosin light chain 9; MRTF-A = myocardin-related transcription factor A; MTP = microsomal triglyceride transfer protein; NOR1 = neuron-derived orphan receptor 1; NOX4 = NADPH oxidase 4; NPC1 = Niemann-Pick C1; OPN = osteopontin; ORP9 = oxysterol binding protein-like 9; PAK1 = p21/Cdc42/Rac1-activated kinase 1; PAPP-A = pregnancy-associated plasma protein A; PDCD4 = programmed cell death 4; PDGF-Rα = platelet-derived growth factor receptor α; PKC-ε = protein kinase C- ε; PIM-1 = serine/threonine-protein kinase PIM-1; PPARα = peroxisome proliferators-activated receptor-α; PPARδ = peroxisome proliferators-activated receptor-δ; PRKAA1 = protein kinase, AMP-activated, alpha 1 catalytic subunit; PTEN = phosphatase and tensin homolog; RUNX2 = Runt-related transcription factor 2; SEMA6A = semaphorin 6A; SIRT1 = sirtuin 1; SirT1 = silent information regulator 1; SMAD1 = SMAD family member 1; SMAD2 = SMAD family member 2; SMAD4 = SMAD family member 4; SOCS5 = suppressor of cytokine signalling 5; SP1 = SP1 transcription factor; SP7 = SP7 transcription factor; SREBP-1 = sterol regulatory element-binding protein 1; SREBP-2 = sterol regulatory element-binding protein 2; STAT5A = signal transducer and activator of transcription 5A; TGFBR1 = transforming growth factor beta receptor 1; THBS1 = thrombospondin 1; TIMP3 = tissue inhibitor of metalloproteinase 3; TLR4 = toll-like receptor 4; TPM1 = tropomyosin 1; TRAF6 = TNF receptor associated factor 6; VCAM-1 = vascular cell adhesion molecule 1; XIAP = X-linked inhibitor of apoptotic protein; βTRC = β-transducin repeat-containing

Table S2: Summary of papers assessing the role of circulating microRNAs as biomarkers in the diagnosis of acute coronary syndromes

Study Population	Source	Notable miRNAs	Ref
AMI = 348; CAD = 100; HC = 368	Plasma	↓ = miR-125b, miR320b	[111]
ACS = 89; CAD = 86; HC = 103	Serum	↑ = miR-146a, miR-186, miR-208b, miR-499	[112]
AMI = 224; Non-AMI CP = 931	Plasma	↑ = miR-208b, miR-320a, miR-499<br>↔ = miR-133a, miR-223, miR-451	[113]
AMI = 17; CAD = 4; HC = 5	Plasma	↑ = miR-423-5p<br>↔ = miR-1, miR-208a	[114]
UA = 19; AP = 34; HC = 20	Plasma	↑ = miR-1, miR-122, miR-126, miR-133a, miR-133b, miR-145, miR-199, miR-433, miR-485-3p<br>↔ = miR-17-5p, miR-92a, miR-155, miR-208a, miR-375, miR-499-5p	[115]
AMI = 13; AP = 176; HC = 127	Plasma	↑ = miR-133a	[116]
AMI = 21; CAD = 17; HC = 30	Plasma	↑ = miR-499	[117]
AMI = 117; AP = 182; HC = 100	Serum	↑ = miR-1, miR-134, miR-186, miR-208, miR-223, miR-499	[118]
STEMI = 173; NSTEMI = 146; Non-AMI CP = 88	Plasma	↑ = miR-1, miR-208b, miR-499-5p	[119]
AMI = 15; HC = 15	Serum	↑ = miR-34a	[120]
AMI = 67; HC = 32	Plasma	↑ = miR-1, miR-133a, miR-208b, miR-499	[121]
AMI = 18; HC = 30	Plasma	↑ = miR-30a, miR-195<br>↓ = let-7b	[122]
AMI = 30; AP = 35; Non-CAD = 22	Plasma	↑ = miR-146a, miR-155<br>↔ = miR-9, miR-125a-5p<br>↓ = miR-29a	[72]
AMI = 17; HC = 25	Plasma	↑ = miR-1<br>↓ = miR-126	[123]
NSTEMI = 92; HF = 81; HC = 99	Plasma	↑ = miR-1, miR-21, miR-133a, miR-423-5p, miR-499-5p<br>↔ = miR-208a	[124]
STEMI = 397; NSTEMI = 113; HC = 87	Plasma	↑ = miR-208b, miR-499	[125]
AMI = 51; HC = 28	Plasma & WB	↑ = miR-133, miR-328	[126]
AMI = 16; UA = 15; AP = 15; NCCP = 16	Plasma	↑ = miR-21, miR-146a<br>↔ = miR-31, miR-125a-5p, miR-125b<br>↓ = miR-155	[127]
AMI = 327; UA = 117	Plasma	↑ = miR-1, miR-133a, miR-208b<br>↔ = miR-133b, miR-208a, miR-499	[128]
STEMI = 25; HC = 11	Plasma	↑ = miR-1, miR-133a, miR-208b, miR-499-5p	[129]
ACS = 29; Non-ACS = 42	Serum	↑ = miR-1, miR-133a	[130]
AMI = 36; Non-AMI CP = 32	Plasma	↑ = miR-1 (NS), miR-133a, miR-208b, and miR-499<br>↔ = miR-122<br>↓ = miR-223	[131]
STEMI = 20; Stable CAD = 20; HC = 20	WB	↑ = miR-30c, miR-145<br>↓ = miR-663b, miR-1291	[132]

Study Population	Source	Notable miRNAs	Ref
STEMI = 33; HC = 17	Plasma	↑ = miR-1, miR-133a, miR-133b, miR-499-5p<br>↔ = miR-208a<br>↓ = miR-122, miR-375	[133]
AMI = 31; HC = 20	Serum	↑ = miR-1	[134]
AMI = 9; UA = 5; HC = 10	Plasma	↑ = miR-499	[135]
UA = 25; AP = 25; HC = 20	PBMC	↑ = miR-134, miR-198 & miR-370	[136]
AMI = 26; UA = 25; AP = 22; NCCP = 18	PBMC	↑ = miR-146a	[137]
AMI = 33; Non-AMI CP = 33; HC = 30	Plasma	↑ = miR-1, miR-133a, miR-208a, miR-499	[138]
AMI = 93; HC = 66	Plasma	↑ = miR-1<br>↔ = miR-133	[139]

ACS = Acute Coronary Syndrome; AMI = Acute Myocardial Infarction; AP = Angina Pectoris; CAD = Coronary Artery Disease; CP = Chest Pain; HC = Healthy Control; HF = Heart Failure; NCCP = Non-Cardiac Chest Pain; NSTEMI = Non–ST-Elevation Myocardial Infarction; PBMC = Peripheral Blood Mononuclear Cells; STEMI = ST-Elevation Myocardial Infarction; UA = Unstable Angina; WB = Whole Blood

Table S3: Summary of studies assessing the role of circulating microRNAs as biomarkers of prognosis following acute coronary syndromes

Study Population	Source	Notable miRNAs	Summary Results	Ref
NSTEMI = 142	Plasma	miR-21, miR-499-5p	Increased miR-499-5p levels at admission were associated with a significant increase in 12-month cardiovascular mortality in multivariate analyses; this association was not present for 24-month mortality<br>The performance of the overall model to predict 12-month mortality was moderate at best	[140]
AMI = 224<br>Non-AMI CP = 931	Plasma	miR-133a, miR-208b, miR-223, miR-320a, miR-451	None of the studied miRNAs predicted long-term mortality or recurrent AMI<br>miR-208b was a moderate predictor of 30 day mortality but not recurrent AMI	[113]
AMI = 150	Plasma	miR-16, miR-27a, miR-101, miR-150	Low levels of miR-101 & miR-150 and high levels of miR-16 & miR-27a were associated with increased risk of impaired LV contractility at 6-months<br>A combination of the miR16, miR-27a, miR-101 & miR-150 improved the prediction of LV contractility based on clinical variables	[141]
AMI = 510	Plasma	miR-208b, miR-499	Increased miR-208b was associated with increased age & increased BMI<br>miR-208b & miR-499 were not associated with diabetes, gender, hypercholesterolaemia or smoking<br>miR-208b & miR-499 were not associated with mortality at any time-point	[142]
AMI = 86	Serum	miR-34a, miR-192, miR-194. miR-208, miR-499	miR-34a, miR-192 & miR-194 were upregulated in patients who developed HF within 1 year of AMI vs. those who did not<br>miR-34a & miR-194 were positively correlated with LV diastolic dimension & negatively correlated with LVEF<br>miR-208 & miR-499 were undetectable in either group	[143]
STEMI = 90	Plasma	miR-150	miR-150 was downregulated in patients with remodelling (ΔLV EDV >0) vs. those without<br>miR-150 outperformed Nt-proBNP to predict remodelling	[144]
STEMI = 173<br>NSTEMI = 146<br>Non-AMI CP = 88	Plasma	miR-1, miR-208b, miR-499-5p	miR-208b & miR-499-5p were associated with death or heart failure in multivariate analyses<br>These associations were lost after adjustment for hsTnT<br>miR-1 was not associated with death or heart failure in any of the analyses<br>All miRNAs were weakly negatively associated with LVEF in multivariate analyses	[119]
AMI = 246	Plasma	miR-133a, miR-423-5p	miR-133a & miR-423-5p were not associated with any markers of LV remodelling in univariate or multivariate analyses<br>miR-133a & miR-423-5p were not associated with peak CK-MB or LVEF at baseline and did not differ between those who did or did not develop heart failure during hospitalisation<br>miR-133a & miR-423-5p were not associated with BNP at any point during 12 month follow-up	[145]
STEMI = 216	Serum	miR-133a	miR-133a was significantly correlated with infarct size, MVO & MSI<br>miR-133a was a univariate predictor of major adverse cardiovascular events but this association was lost upon multivariate adjustment for traditional clinical and CMR prognostic markers	[146]
AMI = 49	Plasma	miR-1	miR-1 levels negatively correlated with LVEF, LV fractional shortening & the ratio of peak early diastolic velocity of mitral flow to late diastolic velocity of mitral flow	[147]
AMI = 40<br>(Death = 19;<br>Survival = 21)	Serum	miR-155, miR-380*	miR-155 & miR380* were significantly increased in the 'cardiac death within 1 year' group vs. survivors	[148]

Study Population	Source	Notable miRNAs	Summary Results	Ref
Prospective Study = 820; AMI = 47	Plasma	miR-126, miR-197, miR-223	Baseline levels of miR-126, miR-197 & miR-223 were associated with risk of AMI at 10 year follow-up<br>Addition of these three miRNAs improved classification in the Framingham Risk Score for hard CAD	[149]
AMI = 12<br>HC = 12	Plasma	miR-1, miR-21, miR-29a, miR-133a, miR-208a	miR-29a on Day 5 post-AMI was significantly associated with LV EDV on Day 90 post-AMI	[150]
AMI = 327<br>UA = 117	Plasma	miR-1, miR-133a, miR-133b, miR-208a, miR-208b, miR-499	miR-133a & miR-208b significantly associated with death in multivariate analyses<br>This association was lost after adjustment for hsTnT	[128]

AMI = Acute Myocardial Infarction; BMI = Body Mass Index; CAD = Coronary Artery Disease; CK-MB = Creatine Kinase-MB Isoenzyme; CMR = Cardiovascular Magnetic Resonance Imaging; CP = Chest Pain; EDV = End Diastolic Volume; HC = Healthy Control; HF = Heart Failure; hsTnT = High Sensitivity Troponin T; LV = Left Ventricle; LVEF = Left Ventricular Ejection Fraction; MSI = Myocardial Salvage Index; MVO = Microvascular Obstruction; NSTEMI = Non–ST-Elevation Myocardial Infarction; Nt-proBNP = N-terminal prohormone of Brain-type Natriuretic Peptide; STEMI = ST-Elevation Myocardial Infarction; UA = Unstable Angina

Table S4: Summary of papers assessing the role of circulating microRNAs as biomarkers in the diagnosis of stable coronary artery disease

Study Population	Source	Notable miRNAs	Ref
AMI = 348; CAD = 100; HC = 368	Plasma	↓ = miR-125b, miR320b	[111]
CAD with Calcified Plaque = 62;<br>CAD with Non-calcified Plaque = 63;<br>Non-CAD = 61	Serum	↓ = miR-21	[151]
CAD = 45; HC = 25	Plasma	↓ = miR-31	[152]
ACS = 89; Stable CAD = 86;<br>Intermediate CV Risk = 40;<br>Low CV Risk = 112; HC = 103	Serum	↑ = miR-146a, miR-186, miR-208b, miR-499	[112]
UA = 58; AP = 31; NCCP = 50	Plasma	↑ = miR-21, miR-25, miR-92a, miR-106b, miR-126*, miR-451, miR-590-5p	[153]
AMI = 17; CAD = 4; HC = 5	Plasma	↔ = miR-1, miR-208a, miR-423-5p	[114]
UA = 19; AP = 34; HC = 20	Plasma	↑ = miR-337-5p, miR-433, miR485-3p<br>miR-1, miR-122, miR-126, miR133a, miR-133b, miR-199a<br>↔ = miR-17-5p, miR-92a, miR-145, miR-155, miR-208a, miR-375, miR-499-5p	[115]
AMI = 13; AP = 176; HC = 127	Plasma	↑ = miR-133a	[116]
AMI = 12; UA = 16; AP = 12;<br>NCCP = 10; HC = 5	Plasma	↓ = miR-214	[154]
AMI = 30; AP = 35; Non-CAD = 22	Plasma	↑ = miR-146a, miR-155<br>↔ = miR-9, miR-125a-5p<br>↓ = miR-29a	[72]
CAD ≥ 70% = 34; CAD < 70% = 49	Plasma	↑ = miR-490-3p, miR-494, miR-769-3p	[155]
CAD = 31; Non-CAD = 36	Plasma	↔ = miR-126	[156]
CAD = 155; Non-CAD = 100	Plasma	↑ = miR-122, miR-370<br>↔ = miR-33a, and miR-33b	[157]
CAD = 81; Non-CAD = 44	$CD14^+$<br>PBMC	↔ = miR-181b, miR-181d<br>↓ = miR-181a	[158]
CAD = 12; HC = 12	WB	↑ = miR-140-3p, miR-182	[159]
CAD = 67; HC = 31	Plasma	↔ = miR-133a, miR208a<br>↓ = miR-17, miR-92a, miR-126, miR-145, miR-155, miR-199a (NS)	[160]
UA = 25; AP = 25; HC = 20	PBMC	↑ = miR-135a<br>↓ = miR-147	[136]

ACS = Acute Coronary Syndrome; AMI = Acute Myocardial Infarction; AP = Angina Pectoris; CAD = Coronary Artery Disease; CV = Cardiovascular; HC = Healthy Control; NCCP = Non-Cardiac Chest Pain; PBMC = Peripheral Blood Mononuclear Cells; UA = Unstable Angina; WB = Whole Blood

Table S5: Summary of papers assessing the role of circulating microRNAs as biomarkers in the diagnosis of heart failure

Study Population	Source	Notable miRNAs	Ref
HF = 53; HC = 39	WB & Serum	↑ = miR-122, miR-200b, miR-519e*, miR-520d-5p, miR-622<br>↓ = miR-558	[161]
HF = 76; COPD = 47; Other SOB = 59; HC = 29	Plasma	↓ = miR-103, miR-142-3p, miR-30b, miR-342-3p	[162]
HF = 22; HC = 18	Serum	↑ = miR-30a, miR-210	[163]
HF = 13; HC = 6	PBMC	↑ = miR-210	[164]
NSTEMI = 92; HF = 81; HC = 99	Plasma	↑ = miR-21, miR-133a, miR-423-5p, miR-499-5p<br>↔ = miR-1, miR-208a	[124]
HF = 30; HC = 30	Serum	↑ = miR-22, miR-92b, miR-320a, miR-423-5p	[165]
HF = 33; HC = 17	Plasma	↔ = miR-122, miR-499<br>↓ = miR-126	[166]
HF = 33; HC = 34	Plasma	↑ = miR-122, miR-133a (NS), miR-208b (NS), miR-499<br>↔ = miR-1, miR-122, miR-233	[131]
NIDCM = 26; ICM = 23; HC = 28	PBMC	↑ = miR-29b, miR-142-3p<br>↓ = miR-107, miR-125b, miR-139, miR-142-5p, miR-497	[167]
HF = 15; HC = 10	Plasma	↔ = miR-499	[135]
HF = 42; Non-HF SOB = 20; HC = 51	Plasma	↑ = miR-18b*, miR-129-5p, miR-423-5p, miR-622, miR-675, miR-1254, HS_202.1	[168]

COPD = Chronic Obstructive Pulmonary Disease; HC = Healthy Control; HF = Heart Failure; ICM = Ischaemic Cardiomyopathy; NIDCM = Non-Ischaemic Dilated Cardiomyopathy; NSTEMI = Non–ST-Elevation Myocardial Infarction; PBMC = Peripheral Blood Mononuclear Cells; SOB = Shortness of Breath; WB = Whole Blood

REFERENCES FOR SUPPLEMENTARY TABLES

1. Wang D, Xia M, Yan X, et al. Gut microbiota metabolism of anthocyanin promotes reverse cholesterol transport in mice via repressing miRNA-10b. *Circ Res* 2012;111:967-81.

2. Sun D, Zhang J, Xie J, et al. MiR-26 controls LXR-dependent cholesterol efflux by targeting ABCA1 and ARL7. *FEBS Lett* 2012;586:1472-9.

3. Zhang M, Wu JF, Chen WJ, et al. MicroRNA-27a/b regulates cellular cholesterol efflux, influx and esterification/hydrolysis in THP-1 macrophages. *Atherosclerosis* 2014;234:54-64.

4. Vickers KC, Shoucri BM, Levin MG, et al. MicroRNA-27b is a regulatory hub in lipid metabolism and is altered in dyslipidemia. *Hepatology* 2013;57:533-42.

5. Soh J, Iqbal J, Queiroz J, et al. MicroRNA-30c reduces hyperlipidemia and atherosclerosis in mice by decreasing lipid synthesis and lipoprotein secretion. *Nat Med* 2013;19:892-900.

6. Sene A, Khan AA, Cox D, et al. Impaired cholesterol efflux in senescent macrophages promotes age-related macular degeneration. *Cell Metab* 2013;17:549-61.

7. Rayner KJ, Suarez Y, Davalos A, et al. MiR-33 contributes to the regulation of cholesterol homeostasis. *Science* 2010;328:1570-3.

8. Marquart TJ, Allen RM, Ory DS, et al. miR-33 links SREBP-2 induction to repression of sterol transporters. *Proc Natl Acad Sci U S A* 2010;107:12228-32.

9. Rayner KJ, Sheedy FJ, Esau CC, et al. Antagonism of miR-33 in mice promotes reverse cholesterol transport and regression of atherosclerosis. *J Clin Invest* 2011;121:2921-31.

10. Horie T, Baba O, Kuwabara Y, et al. MicroRNA-33 deficiency reduces the progression of atherosclerotic plaque in ApoE-/- mice. *J Am Heart Assoc* 2012;1:e003376.

11. Rotllan N, Ramirez CM, Aryal B, et al. Therapeutic silencing of microRNA-33 inhibits the progression of atherosclerosis in Ldlr-/- mice--brief report. *Arterioscler Thromb Vasc Biol* 2013;33:1973-7.

12. Horie T, Nishino T, Baba O, et al. MicroRNA-33 regulates sterol regulatory element-binding protein 1 expression in mice. *Nat Commun* 2013;4:2883.

13. Rayner KJ, Esau CC, Hussain FN, et al. Inhibition of miR-33a/b in non-human primates raises plasma HDL and lowers VLDL triglycerides. *Nature* 2011;478:404-7.

14. Davalos A, Goedeke L, Smibert P, et al. miR-33a/b contribute to the regulation of fatty acid metabolism and insulin signaling. *Proc Natl Acad Sci U S A* 2011;108:9232-7.

15. Kim J, Yoon H, Ramirez CM, et al. MiR-106b impairs cholesterol efflux and increases Aβ levels by repressing ABCA1 expression. *Exp Neurol* 2012;235:476-83.

16. Ramirez CM, Rotllan N, Vlassov AV, et al. Control of cholesterol metabolism and plasma high-density lipoprotein levels by microRNA-144. *Circ Res* 2013;112:1592-601.

17. Hu YW, Hu YR, Zhao JY, et al. An Agomir of miR-144-3p Accelerates Plaque Formation through Impairing Reverse Cholesterol Transport and Promoting Pro-Inflammatory Cytokine Production. *PLoS One* 2014;9:e94997.

18. Yang M, Liu W, Pellicane C, et al. Identification of miR-185 as a regulator of de novo cholesterol biosynthesis and low density lipoprotein uptake. *J Lipid Res* 2014;55:226-38.

19. Vinod M, Chennamsetty I, Colin S, et al. miR-206 controls LXRα expression and promotes LXR-mediated cholesterol efflux in macrophages. *Biochim Biophys Acta* 2014;1841:827-35.

20. Wang D, Yan X, Xia M, et al. Coenzyme Q10 Promotes Macrophage Cholesterol Efflux by Regulation of the Activator Protein-1/MicroRNA-378/ATP-Binding Cassette Transporter G1-Signaling Pathway. *Arterioscler Thromb Vasc Biol* 2014;34:1860-70.

21. Tian GP, Tang YY, He PP, et al. The effects of miR-467b on lipoprotein lipase (LPL) expression, pro-inflammatory cytokine, lipid levels and atherosclerotic lesions in apolipoprotein E knockout mice. *Biochem Biophys Res Commun* 2014;443:428-34.

22. Tian GP, Chen WJ, He PP, et al. MicroRNA-467b targets LPL gene in RAW 264.7 macrophages and attenuates lipid accumulation and proinflammatory cytokine secretion. *Biochimie* 2012;94:2749-55.

23. Zhao R, Feng J, He G. miR-613 regulates cholesterol efflux by targeting LXRα and ABCA1 in PPARγ activated THP-1 macrophages. *Biochem Biophys Res Commun* 2014;448:329-34.

24. Ramirez CM, Davalos A, Goedeke L, et al. MicroRNA-758 regulates cholesterol efflux through posttranscriptional repression of ATP-binding cassette transporter A1. *Arterioscler Thromb Vasc Biol* 2011;31:2707-14.

25. Wang H, Zhu HQ, Wang F, et al. MicroRNA-1 prevents high-fat diet-induced endothelial permeability in apoE knock-out mice. *Mol Cell Biochem* 2013;378:153-9.

26. Urbich C, Kaluza D, Fromel T, et al. MicroRNA-27a/b controls endothelial cell repulsion and angiogenesis by targeting semaphorin 6A. *Blood* 2012;119:1607-16.

27. Ito T, Yagi S, Yamakuchi M. MicroRNA-34a regulation of endothelial senescence. *Biochem Biophys Res Commun* 2010;398:735-40.

28. Wu W, Xiao H, Laguna-Fernandez A, et al. Flow-Dependent Regulation of Kruppel-Like Factor 2 Is Mediated by MicroRNA-92a. *Circulation* 2011;124:633-41.

29. Fang Y, Davies PF. Site-specific microRNA-92a regulation of Kruppel-like factors 4 and 2 in atherosusceptible endothelium. *Arterioscler Thromb Vasc Biol* 2012;32:979-87.

30. Loyer X, Potteaux S, Vion AC, et al. Inhibition of microRNA-92a prevents endothelial dysfunction and atherosclerosis in mice. *Circ Res* 2014;114:434-43.

31. Zhang L, Zhou M, Qin G, et al. MiR-92a regulates viability and angiogenesis of endothelial cells under oxidative stress. *Biochem Biophys Res Commun* 2014;446:952-8.

32. Schober A, Nazari-Jahantigh M, Wei Y, et al. MicroRNA-126-5p promotes endothelial proliferation and limits atherosclerosis by suppressing Dlk1. *Nat Med* 2014;20:368-76.

33. Fu X, Huang X, Li P, et al. 7-Ketocholesterol inhibits isocitrate dehydrogenase 2 expression and impairs endothelial function via microRNA-144. *Free Radic Biol Med* 2014;71:1-15.

34. Vasa-Nicotera M, Chen H, Tucci P, et al. miR-146a is modulated in human endothelial cell with aging. *Atherosclerosis* 2011;217:326-30.

35. Weber M, Kim S, Patterson N, et al. MiRNA-155 targets myosin light chain kinase and modulates actin cytoskeleton organization in endothelial cells. *Am J Physiol Heart Circ Physiol* 2014;306:H1192-203.

36. Zhu N, Zhang D, Chen S, et al. Endothelial enriched microRNAs regulate angiotensin II-induced endothelial inflammation and migration. *Atherosclerosis* 2011;215:286-93.

37. Menghini R, Casagrande V, Marino A, et al. MiR-216a: a link between endothelial dysfunction and autophagy. *Cell Death Dis* 2014;5:e1029.

38. Menghini R, Casagrande V, Cardellini M, et al. MicroRNA 217 modulates endothelial cell senescence via silent information regulator 1. *Circulation* 2009;120:1524-32.

39. Zhang X, Mao H, Chen JY, et al. Increased expression of microRNA-221 inhibits PAK1 in endothelial progenitor cells and impairs its function via c-Raf/MEK/ERK pathway. *Biochem Biophys Res Commun* 2013;431:404-8.

40. Poliseno L, Tuccoli A, Mariani L, et al. MicroRNAs modulate the angiogenic properties of HUVECs. *Blood* 2006;108:3068-71.

41. Dentelli P, Rosso A, Orso F, et al. microRNA-222 controls neovascularization by regulating signal transducer and activator of transcription 5A expression. *Arterioscler Thromb Vasc Biol* 2010;30:1562-8.

42. Suarez Y, Fernandez-Hernando C, Pober JS, et al. Dicer dependent microRNAs regulate gene expression and functions in human endothelial cells. *Circ Res* 2007;100:1164-73.

43. Liu X, Cheng Y, Yang J, et al. Cell-specific effects of miR-221/222 in vessels: molecular mechanism and therapeutic application. *J Mol Cell Cardiol* 2012;52:245-55.

44. Pan Y, Liang H, Liu H, et al. Platelet-secreted microRNA-223 promotes endothelial cell apoptosis induced by advanced glycation end products via targeting the insulin-like growth factor 1 receptor. *J Immunol* 2014;192:437-46.

45. Qin B, Xiao B, Liang D, et al. MicroRNAs expression in ox-LDL treated HUVECs: MiR-365 modulates apoptosis and Bcl-2 expression. *Biochem Biophys Res Commun* 2011;410:127-33.

46. Ying C, Sui-Xin L, Kang-Ling X, et al. MicroRNA-492 reverses high glucose-induced insulin resistance in HUVEC cells through targeting resistin. *Mol Cell Biochem* 2014;391:117-25.

47. Shin S, Moon KC, Park KU, et al. MicroRNA-513a-5p mediates TNF-α and LPS induced apoptosis via downregulation of X-linked inhibitor of apoptotic protein in endothelial cells. *Biochimie* 2012;94:1431-6.

48. Ni CW, Qiu H, Jo H. MicroRNA-663 upregulated by oscillatory shear stress plays a role in inflammatory response of endothelial cells. *Am J Physiol Heart Circ Physiol* 2011;300:H1762-9.

49. Son DJ, Kumar S, Takabe W, et al. The atypical mechanosensitive microRNA-712 derived from pre-ribosomal RNA induces endothelial inflammation and atherosclerosis. *Nat Commun* 2013;4:3000.

50. Qin B, Xiao B, Liang D, et al. MicroRNA let-7c inhibits Bcl-xl expression and regulates ox-LDL-induced endothelial apoptosis. *BMB Rep* 2012;45:464-9.

51. Zhang Y, Chen N, Zhang J, et al. Hsa-let-7g miRNA targets caspase-3 and inhibits the apoptosis induced by ox-LDL in endothelial cells. *Int J Mol Sci* 2013;14:22708-20.

52. Liao YC, Wang YS, Guo YC, et al. Let-7g Improves Multiple Endothelial Functions Through Targeting Transforming Growth Factor-Beta and SIRT-1 Signaling. *J Am Coll Cardiol* 2014;63:1685-94.

53. Thulin P, Wei T, Werngren O, et al. MicroRNA-9 regulates the expression of peroxisome proliferator-activated receptor δ in human monocytes during the inflammatory response. *Int J Mol Med* 2013;31:1003-10.

54. Xu J, Hu G, Lu M, et al. MiR-9 reduces human acyl-coenzyme A:cholesterol acyltransferase-1 to decrease THP-1 macrophage-derived foam cell formation. *Acta Biochim. Biophys. Sin. (Shanghai)* 2013;45:953-62.

55. Fang Y, Shi C, Manduchi E, et al. MicroRNA-10a regulation of proinflammatory phenotype in athero-susceptible endothelium in vivo and in vitro. *Proc Natl Acad Sci U S A* 2010;107:13450-5.

56. Liu X, Wang L, Li H, et al. Coactivator-associated arginine methyltransferase 1 targeted by miR-15a regulates inflammation in acute coronary syndrome. *Atherosclerosis* 2014;233:349-56.

57. Suarez Y, Wang C, Manes TD, et al. Cutting edge: TNF-induced microRNAs regulate TNF-induced expression of E-selectin and intercellular adhesion molecule-1 on human endothelial cells: feedback control of inflammation. *J Immunol* 2010;184:21-5.

58. Zhou J, Wang KC, Wu W, et al. MicroRNA-21 targets peroxisome proliferators-activated receptor-alpha in an autoregulatory loop to modulate flow-induced endothelial inflammation. *Proc Natl Acad Sci U S A* 2011;108:10355-60.

59. Feng J, Li A, Deng J, et al. miR-21 attenuates lipopolysaccharide-induced lipid accumulation and inflammatory response: potential role in cerebrovascular disease. *Lipids Health Dis* 2014;13:27.

60. Chen T, Li Z, Tu J, et al. MicroRNA-29a regulates pro-inflammatory cytokine secretion and scavenger receptor expression by targeting LPL in oxLDL-stimulated dendritic cells. *FEBS Lett* 2011;585:657-63.

61. Chen T, Huang Z, Wang L, et al. MicroRNA-125a-5p partly regulates the inflammatory response, lipid uptake, and ORP9 expression in oxLDL-stimulated monocyte/macrophages. *Cardiovasc Res* 2009;83:131-9.

62. Harris TA, Yamakuchi M, Ferlito M, et al. MicroRNA-126 regulates endothelial expression of vascular cell adhesion molecule 1. *Proc Natl Acad Sci U S A* 2008;105:1516-21.

63. Sun C, Alkhoury K, Wang YI, et al. IRF-1 and miRNA126 modulate VCAM-1 expression in response to a high-fat meal. *Circ Res* 2012;111:1054-64.

64. Schmitt MM, Megens RT, Zernecke A, et al. Endothelial junctional adhesion molecule-a guides monocytes into flow-dependent predilection sites of atherosclerosis. *Circulation* 2014;129:66-76.

65. Cheng HS, Sivachandran N, Lau A, et al. MicroRNA-146 represses endothelial activation by inhibiting pro-inflammatory pathways. *EMBO Mol Med* 2013;5:949-66.

66. Chen T, Li Z, Jing T, et al. MicroRNA-146a regulates the maturation process and pro-inflammatory cytokine secretion by targeting CD40L in oxLDL-stimulated dendritic cells. *FEBS Lett* 2011;585:567-73.

67. Yang K, He YS, Wang XQ, et al. MiR-146a inhibits oxidized low-density lipoprotein-induced lipid accumulation and inflammatory response via targeting toll-like receptor 4. *FEBS Lett* 2011;585:854-60.

68. Liu G, Friggeri A, Yang Y, et al. miR-147, a microRNA that is induced upon Toll-like receptor stimulation, regulates murine macrophage inflammatory responses. *Proc Natl Acad Sci U S A* 2009;106:15819-24.

69. Tian FJ, An LN, Wang GK, et al. Elevated microRNA-155 promotes foam cell formation by targeting HBP1 in atherogenesis. *Cardiovasc Res* 2014;103:100-10.

70. Nazari-Jahantigh M, Wei Y, Noels H, et al. MicroRNA-155 promotes atherosclerosis by repressing Bcl6 in macrophages. *J Clin Invest* 2012;122:4190-202.

71. Zhu GF, Yang LX, Guo RW, et al. miR-155 inhibits oxidized low-density lipoprotein-induced apoptosis of RAW264.7 cells. *Mol Cell Biochem* 2013;382:253-61.

72. Zhu J, Chen T, Yang L, et al. Regulation of microRNA-155 in atherosclerotic inflammatory responses by targeting MAP3K10. *PLoS One* 2012;7:e46551.

73. Du F, Yu F, Wang Y, et al. MicroRNA-155 deficiency results in decreased macrophage inflammation and attenuated atherogenesis in apolipoprotein E-deficient mice. *Arterioscler Thromb Vasc Biol* 2014;34:759-67.

74. O'Connell RM, Taganov KD, Boldin MP, et al. MicroRNA-155 is induced during the macrophage inflammatory response. *Proc Natl Acad Sci U S A* 2007;104:1604-9.

75. Wu C, Gong Y, Yuan J, et al. microRNA-181a represses ox-LDL-stimulated inflammatory response in dendritic cell by targeting c-Fos. *J Lipid Res* 2012;53:2355-63.

76. Sun X, He S, Wara AK, et al. Systemic delivery of microRNA-181b inhibits nuclear factor-κB activation, vascular inflammation, and atherosclerosis in apolipoprotein E-deficient mice. *Circ Res* 2014;114:32-40.

77. Wei Y, Nazari-Jahantigh M, Chan L, et al. The microRNA-342-5p fosters inflammatory macrophage activation through an Akt1- and microRNA-155-dependent pathway during atherosclerosis. *Circulation* 2013;127:1609-19.

78. Xie C, Huang H, Sun X, et al. MicroRNA-1 regulates smooth muscle cell differentiation by repressing Kruppel-like factor 4. *Stem Cells Dev* 2011;20:205-10.

79. Minami T, Kuwahara K, Nakagawa Y, et al. Reciprocal expression of MRTF-A and myocardin is crucial for pathological vascular remodelling in mice. *EMBO J* 2012;31:4428-40.

80. Chen J, Yin H, Jiang Y, et al. Induction of microRNA-1 by myocardin in smooth muscle cells inhibits cell proliferation. *Arterioscler Thromb Vasc Biol* 2011;31:368-75.

81. Ji R, Cheng Y, Yue J, et al. MicroRNA expression signature and antisense-mediated depletion reveal an essential role of MicroRNA in vascular neointimal lesion formation. *Circ Res* 2007;100:1579-88.

82. Davis BN, Hilyard AC, Lagna G, et al. SMAD proteins control DROSHA-mediated microRNA maturation. *Nature* 2008;454:56-61.

83. Sarkar J, Gou D, Turaka P, et al. MicroRNA-21 plays a role in hypoxia-mediated pulmonary artery smooth muscle cell proliferation and migration. *Am J Physiol Lung Cell Mol Physiol* 2010;299:L861-71.

84. Wang M, Li W, Chang GQ, et al. MicroRNA-21 regulates vascular smooth muscle cell function via targeting tropomyosin 1 in arteriosclerosis obliterans of lower extremities. *Arterioscler Thromb Vasc Biol* 2011;31:2044-53.

85. Leeper NJ, Raiesdana A, Kojima Y, et al. MicroRNA-26a is a novel regulator of vascular smooth muscle cell function. *J Cell Physiol* 2011;226:1035-43.

86. Chen KC, Wang YS, Hu CY, et al. OxLDL up-regulates microRNA-29b, leading to epigenetic modifications of MMP-2/MMP-9 genes: a novel mechanism for cardiovascular diseases. *FASEB J* 2011;25:1718-28.

87. Goettsch C, Rauner M, Pacyna N, et al. miR-125b regulates calcification of vascular smooth muscle cells. *Am J Pathol* 2011;179:1594-600.

88. Zhou J, Li YS, Nguyen P, et al. Regulation of vascular smooth muscle cell turnover by endothelial cell-secreted microRNA-126: role of shear stress. *Circ Res* 2013;113:40-51.

89. Choe N, Kwon JS, Kim JR, et al. The microRNA miR-132 targets Lrrfip1 to block vascular smooth muscle cell proliferation and neointimal hyperplasia. *Atherosclerosis* 2013;229:348-55.

90. Torella D, Iaconetti C, Catalucci D, et al. MicroRNA-133 controls vascular smooth muscle cell phenotypic switch in vitro and vascular remodeling in vivo. *Circ Res* 2011;109:880-93.

91. Liao XB, Zhang ZY, Yuan K, et al. MiR-133a modulates osteogenic differentiation of vascular smooth muscle cells. *Endocrinology* 2013;154:3344-52.

92. Gao S, Wassler M, Zhang L, et al. MicroRNA-133a regulates insulin-like growth factor-1 receptor expression and vascular smooth muscle cell proliferation in murine atherosclerosis. *Atherosclerosis* 2014;232:171-9.

93. Cordes KR, Sheehy NT, White MP, et al. miR-145 and miR-143 regulate smooth muscle cell fate and plasticity. *Nature* 2009;460:705-10.

94. Xin M, Small EM, Sutherland LB, et al. MicroRNAs miR-143 and miR-145 modulate cytoskeletal dynamics and responsiveness of smooth muscle cells to injury. *Genes Dev* 2009;23:2166-78.

95. Boettger T, Beetz N, Kostin S, et al. Acquisition of the contractile phenotype by murine arterial smooth muscle cells depends on the Mir143/145 gene cluster. *J Clin Invest* 2009;119:2634-47.

96. Quintavalle M, Elia L, Condorelli G, et al. MicroRNA control of podosome formation in vascular smooth muscle cells in vivo and in vitro. *J Cell Biol* 2010;189:13-22.

97. Hergenreider E, Heydt S, Treguer K, et al. Atheroprotective communication between endothelial cells and smooth muscle cells through miRNAs. *Nat Cell Biol* 2012;14:249-56.

98. Cheng Y, Liu X, Yang J, et al. MicroRNA-145, a novel smooth muscle cell phenotypic marker and modulator, controls vascular neointimal lesion formation. *Circ Res* 2009;105:158-66.

99. Lovren F, Pan Y, Quan A, et al. MicroRNA-145 targeted therapy reduces atherosclerosis. *Circulation* 2012;126:S81-90.

100. Remus EW, Lyle AN, Weiss D, et al. miR181a protects against angiotensin II-induced osteopontin expression in vascular smooth muscle cells. *Atherosclerosis* 2013;228:168-74.

101. Wang YS, Wang HY, Liao YC, et al. MicroRNA-195 regulates vascular smooth muscle cell phenotype and prevents neointimal formation. *Cardiovasc Res* 2012;95:517-26.

102. Zhang Y, Wang Y, Wang X, et al. Insulin promotes vascular smooth muscle cell proliferation via microRNA-208-mediated downregulation of p21. *J Hypertens* 2011;29:1560-8.

103. Kothapalli D, Castagnino P, Rader DJ, et al. Apolipoprotein E-mediated cell cycle arrest linked to p27 and the Cox2-dependent repression of miR221/222. *Atherosclerosis* 2013;227:65-71.

104. Davis BN, Hilyard AC, Nguyen PH, et al. Induction of microRNA-221 by platelet-derived growth factor signaling is critical for modulation of vascular smooth muscle phenotype. *J Biol Chem* 2009;284:3728-38.

105. Liu X, Cheng Y, Zhang S, et al. A necessary role of miR-221 and miR-222 in vascular smooth muscle cell proliferation and neointimal hyperplasia. *Circ Res* 2009;104:476-87.

106. Sun Y, Chen D, Cao L, et al. MiR-490-3p modulates the proliferation of vascular smooth muscle cells induced by ox-LDL through targeting PAPP-A. *Cardiovasc Res* 2013;100:272-9.

107. Li P, Liu Y, Yi B, et al. MicroRNA-638 is highly expressed in human vascular smooth muscle cells and inhibits PDGF-BB-induced cell proliferation and migration through targeting orphan nuclear receptor NOR1. *Cardiovasc Res* 2013;99:185-93.

108. Li P, Zhu N, Yi B, et al. MicroRNA-663 regulates human vascular smooth muscle cell phenotypic switch and vascular neointimal formation. *Circ Res* 2013;113:1117-27.

109. Yu ML, Wang JF, Wang GK, et al. Vascular smooth muscle cell proliferation is influenced by let-7d microRNA and its interaction with KRAS. *Circ J* 2011;75:703-9.

110. Chen KC, Hsieh IC, Hsi E, et al. Negative feedback regulation between microRNA let-7g and the oxLDL receptor LOX-1. *J Cell Sci* 2011;124:4115-24.

111. Huang S, Chen M, Li L, et al. Circulating microRNAs and the Occurrence of Acute Myocardial Infarction in Chinese Populations. *Circ Cardiovasc Genet* 2014;7:189-98.

112. Wu J, Song J, Wang C, et al. Identification of serum microRNAs for cardiovascular risk stratification in dyslipidemia subjects. *Int J Cardiol* 2014;172:232-4.

113. Devaux Y, Mueller M, Haaf P, et al. Diagnostic and prognostic value of circulating microRNAs in patients with acute chest pain. *J Intern Med* Published Online First: 17 December 2013. doi:10.1111/joim.12183.

114. Nabialek E, Wanha W, Kula D, et al. Circulating microRNAs (miR-423-5p, miR-208a and miR-1) in acute myocardial infarction and stable coronary heart disease. *Minerva Cardioangiol* 2013;61:627-37.

115. D'Alessandra Y, Carena MC, Spazzafumo L, et al. Diagnostic potential of plasmatic MicroRNA signatures in stable and unstable angina. *PLoS One* 2013;8:e80345.

116. Wang F, Long G, Zhao C, et al. Plasma microRNA-133a is a new marker for both acute myocardial infarction and underlying coronary artery stenosis. *J Transl Med* 2013;11:222.

117. Zheng W, Di Y, Liu Y, et al. Development and application of a novel reverse transcription real-time PCR method for miR-499 quantification. *Clin Biochem* 2013;46:1566-71.

118. Li C, Fang Z, Jiang T, et al. Serum microRNAs profile from genome-wide serves as a fingerprint for diagnosis of acute myocardial infarction and angina pectoris. *BMC Med Genomics* 2013;6:16.

119. Gidlof O, Smith JG, Miyazu K, et al. Circulating cardio-enriched microRNAs are associated with long-term prognosis following myocardial infarction. *BMC Cardiovasc Disord* 2013;13:12.

120. Fan F, Sun A, Zhao H, et al. MicroRNA-34a promotes cardiomyocyte apoptosis post myocardial infarction through down-regulating aldehyde dehydrogenase 2. *Curr Pharm Des* 2013;19:4865-73.

121. Li YQ, Zhang MF, Wen HY, et al. Comparing the diagnostic values of circulating microRNAs and cardiac troponin T in patients with acute myocardial infarction. *Clinics (Sao Paulo)* 2013;68:75-80.

122. Long G, Wang F, Duan Q, et al. Circulating miR-30a, miR-195 and let-7b associated with acute myocardial infarction. *PLoS One* 2012;7:e50926.

123. Long G, Wang F, Duan Q, et al. Human circulating microRNA-1 and microRNA-126 as potential novel indicators for acute myocardial infarction. *Int J Biol Sci* 2012;8:811-8.

124. Olivieri F, Antonicelli R, Lorenzi M, et al. Diagnostic potential of circulating miR-499-5p in elderly patients with acute non ST-elevation myocardial infarction. *Int J Cardiol* 2013;167:531-6.

125. Devaux Y, Vausort M, Goretti E, et al. Use of circulating microRNAs to diagnose acute myocardial infarction. *Clin Chem* 2012;58:559-67.

126. Wang R, Li N, Zhang Y, et al. Circulating microRNAs are promising novel biomarkers of acute myocardial infarction. *Intern Med* 2011;50:1789-95.

127. Yao R, Ma Y, Du Y, et al. The altered expression of inflammation-related microRNAs with microRNA-155 expression correlates with Th17 differentiation in patients with acute coronary syndrome. *Cell Mol Immunol* 2011;8:486-95.

128. Widera C, Gupta SK, Lorenzen JM, et al. Diagnostic and prognostic impact of six circulating microRNAs in acute coronary syndrome. *J Mol Cell Cardiol* 2011;51:872-5.

129. Gidlof O, Andersson P, van der Pals J, et al. Cardiospecific microRNA plasma levels correlate with troponin and cardiac function in patients with ST elevation myocardial infarction, are selectively dependent on renal elimination, and can be detected in urine samples. *Cardiology* 2011;118:217-26.

130. Kuwabara Y, Ono K, Horie T, et al. Increased microRNA-1 and microRNA-133a levels in serum of patients with cardiovascular disease indicate myocardial damage. *Circ Cardiovasc Genet* 2011;4:446-54.

131. Corsten MF, Dennert R, Jochems S, et al. Circulating MicroRNA-208b and MicroRNA-499 reflect myocardial damage in cardiovascular disease. *Circ Cardiovasc Genet* 2010;3:499-506.

132. Meder B, Keller A, Vogel B, et al. MicroRNA signatures in total peripheral blood as novel biomarkers for acute myocardial infarction. *Basic Res Cardiol* 2011;106:13-23.

133. D'Alessandra Y, Devanna P, Limana F, et al. Circulating microRNAs are new and sensitive biomarkers of myocardial infarction. *Eur Heart J* 2010;31:2765-73.

134. Cheng Y, Tan N, Yang J, et al. A translational study of circulating cell-free microRNA-1 in acute myocardial infarction. *Clin Sci (Lond)* 2010;119:87-95.

135. Adachi T, Nakanishi M, Otsuka Y, et al. Plasma microRNA 499 as a biomarker of acute myocardial infarction. *Clin Chem* 2010;56:1183-5.

136. Hoekstra M, van der Lans CA, Halvorsen B, et al. The peripheral blood mononuclear cell microRNA signature of coronary artery disease. *Biochem Biophys Res Commun* 2010;394:792-7.

137. Guo M, Mao X, Ji Q, et al. miR-146a in PBMCs modulates Th1 function in patients with acute coronary syndrome. *Immunol Cell Biol* 2010;88:555-64.

138. Wang GK, Zhu JQ, Zhang JT, et al. Circulating microRNA: a novel potential biomarker for early diagnosis of acute myocardial infarction in humans. *Eur Heart J* 2010;31:659-66.

139. Ai J, Zhang R, Li Y, et al. Circulating microRNA-1 as a potential novel biomarker for acute myocardial infarction. *Biochem Biophys Res Commun* 2010;391:73-7.

140. Olivieri F, Antonicelli R, Spazzafumo L, et al. Admission levels of circulating miR-499-5p and risk of death in elderly patients after acute non-ST elevation myocardial infarction. *Int J Cardiol* 2014;172:e276-8.

141. Devaux Y, Vausort M, McCann GP, et al. A panel of 4 microRNAs facilitates the prediction of left ventricular contractility after acute myocardial infarction. *PLoS One* 2013;8:e70644.

142. Goretti E, Vausort M, Wagner DR, et al. Association between circulating microRNAs, cardiovascular risk factors and outcome in patients with acute myocardial infarction. *Int J Cardiol* 2013;168:4548-50.

143. Matsumoto S, Sakata Y, Suna S, et al. Circulating p53-responsive microRNAs are predictive indicators of heart failure after acute myocardial infarction. *Circ Res* 2013;113:322-6.

144. Devaux Y, Vausort M, McCann GP, et al. MicroRNA-150: a novel marker of left ventricular remodeling after acute myocardial infarction. *Circ Cardiovasc Genet* 2013;6:290-8.

145. Bauters C, Kumarswamy R, Holzmann A, et al. Circulating miR-133a and miR-423-5p fail as biomarkers for left ventricular remodeling after myocardial infarction. *Int J Cardiol* 2013;168:1837-40.

146. Eitel I, Adams V, Dieterich P, et al. Relation of circulating MicroRNA-133a concentrations with myocardial damage and clinical prognosis in ST-elevation myocardial infarction. *Am Heart J* 2012;164:706-14.

147. Zhang R, Niu H, Ban T, et al. Elevated plasma microRNA-1 predicts heart failure after acute myocardial infarction. *Int J Cardiol* 2013;166:259-60.

148. Matsumoto S, Sakata Y, Nakatani D, et al. A subset of circulating microRNAs are predictive for cardiac death after discharge for acute myocardial infarction. *Biochem Biophys Res Commun* 2012;427:280-4.

149. Zampetaki A, Willeit P, Tilling L, et al. Prospective study on circulating microRNAs and risk of myocardial infarction. *J Am Coll Cardiol* 2012;60:290-9.

150. Zile MR, Mehurg SM, Arroyo JE, et al. Relationship between the temporal profile of plasma microRNA and left ventricular remodeling in patients after myocardial infarction. *Circ Cardiovasc Genet* 2011;4:614-9.

151. Fan X, Wang E, Wang X, et al. MicroRNA-21 is a unique signature associated with coronary plaque instability in humans by regulating matrix metalloproteinase-9 via reversion-inducing cysteine-rich protein with Kazal motifs. *Exp Mol Pathol* 2014;96:242-49.

152. Wang HW, Huang TS, Lo HH, et al. Deficiency of the MicroRNA-31-MicroRNA-720 Pathway in the Plasma and Endothelial Progenitor Cells From Patients With Coronary Artery Disease. *Arterioscler Thromb Vasc Biol* 2014.

153. Ren J, Zhang J, Xu N, et al. Signature of circulating microRNAs as potential biomarkers in vulnerable coronary artery disease. *PLoS One* 2013;8:e80738.

154. Lu HQ, Liang C, He ZQ, et al. Circulating miR-214 is associated with the severity of coronary artery disease. *J Geriatr Cardiol* 2013;10:34-8.

155. Freedman JE, Ercan B, Morin KM, et al. The distribution of circulating microRNA and their relation to coronary disease. *F1000Res* 2012;1:50.

156. Sun X, Zhang M, Sanagawa A, et al. Circulating microRNA-126 in patients with coronary artery disease: correlation with LDL cholesterol. *Thromb J* 2012;10:16.

157. Gao W, He HW, Wang ZM, et al. Plasma levels of lipometabolism-related miR-122 and miR-370 are increased in patients with hyperlipidemia and associated with coronary artery disease. *Lipids Health Dis* 2012;11:55.

158. Hulsmans M, Sinnaeve P, Van der Schueren B, et al. Decreased miR-181a expression in monocytes of obese patients is associated with the occurrence of metabolic syndrome and coronary artery disease. *J Clin Endocrinol Metab* 2012;97:E1213-8.

159. Taurino C, Miller WH, McBride MW, et al. Gene expression profiling in whole blood of patients with coronary artery disease. *Clin Sci (Lond)* 2010;119:335-43.

160. Fichtlscherer S, De Rosa S, Fox H, et al. Circulating microRNAs in patients with coronary artery disease. *Circ Res* 2010;107:677-84.

161. Vogel B, Keller A, Frese KS, et al. Multivariate miRNA signatures as biomarkers for non-ischaemic systolic heart failure. *Eur Heart J* 2013;34:2812-22.

162. Ellis KL, Cameron VA, Troughton RW, et al. Circulating microRNAs as candidate markers to distinguish heart failure in breathless patients. *Eur J Heart Fail* 2013;15:1138-47.

163. Zhao DS, Chen Y, Jiang H, et al. Serum miR-210 and miR-30a expressions tend to revert to fetal levels in Chinese adult patients with chronic heart failure. *Cardiovasc Pathol* 2013;22:444-50.

164. Endo K, Naito Y, Ji X, et al. MicroRNA 210 as a biomarker for congestive heart failure. *Biol Pharm Bull* 2013;36:48-54.

165. Goren Y, Kushnir M, Zafrir B, et al. Serum levels of microRNAs in patients with heart failure. *Eur J Heart Fail* 2012;14:147-54.

166. Fukushima Y, Nakanishi M, Nonogi H, et al. Assessment of plasma miRNAs in congestive heart failure. *Circ J* 2011;75:336-40.

167. Voellenkle C, van Rooij J, Cappuzzello C, et al. MicroRNA signatures in peripheral blood mononuclear cells of chronic heart failure patients. *Physiol Genomics* 2010;42:420-6.

168. Tijsen AJ, Creemers EE, Moerland PD, et al. MiR423-5p as a circulating biomarker for heart failure. *Circ Res* 2010;106:1035-9.
